# Single-cell sequencing reveals the immune microenvironment landscape related to anti-PD-1 resistance in metastatic colorectal cancer with high microsatellite instability

**DOI:** 10.1186/s12916-023-02866-y

**Published:** 2023-04-27

**Authors:** Tao Wu, Xuan Zhang, Xinxing Liu, Xinyi Cai, Tao Shen, Dingguo Pan, Rui Liang, Rong Ding, Ruixi Hu, Jianhua Dong, Furong Li, Jinsha Li, Lin Xie, Chunlong Wang, Shilei Geng, Zhaoyu Yang, Lu Xing, YunFeng Li

**Affiliations:** 1 Department of Colorectal Surgery, Yunnan Cancer Hospital, The Third Affiliated Hospital of Kunming Medical University, No. 519, Kunzhou Road, Xishan District, Kunming, 650118 China; 2 College of Bioengineering, Chongqing University, Chongqing, China; 3 Department of Minimally Invasive Intervention, Yunnan Cancer Hospital, The Third Affiliated Hospital of Kunming Medical University, Kunming, China; 4 Department of Gastroenteroscopy, Yunnan Cancer Hospital, The Third Affiliated Hospital of Kunming Medical University, Kunming, China; 5 Department of Oncology, Yunnan Cancer Hospital, The Third Affiliated Hospital of Kunming Medical University, Kunming, China; 6 Department of Radiology, Yunnan Cancer Hospital, The Third Affiliated Hospital of Kunming Medical University, Kunming, China; 7 Department of Dermatology, Kunming Children’s Hospital, Kunming, China

**Keywords:** Metastatic colorectal cancer (mCRC), Programmed cell death protein-1(PD-1), Interleukin-1 beta (IL-1β), Myeloid-derived suppressor cells (MDSCs), microsatellite instability-high (MSI-H), Deficient mismatch repair (dMMR)

## Abstract

**Background:**

The objective response rate of microsatellite instability-high (MSI-H) metastatic colorectal cancer (mCRC) patients with first-line anti-programmed cell death protein-1 (PD-1) monotherapy is only 40–45%. Single-cell RNA sequencing (scRNA-seq) enables unbiased analysis of the full variety of cells comprising the tumor microenvironment. Thus, we used scRNA-seq to assess differences among microenvironment components between therapy-resistant and therapy-sensitive groups in MSI-H/mismatch repair-deficient (dMMR) mCRC. Resistance-related cell types and genes identified by this analysis were subsequently verified in clinical samples and mouse models to further reveal the molecular mechanism of anti-PD-1 resistance in MSI-H or dMMR mCRC.

**Methods:**

The response of primary and metastatic lesions to first-line anti-PD-1 monotherapy was evaluated by radiology. Cells from primary lesions of patients with MSI-H/dMMR mCRC were analyzed using scRNA-seq. To identify the marker genes in each cluster, distinct cell clusters were identified and subjected to subcluster analysis. Then, a protein‒protein interaction network was constructed to identify key genes. Immunohistochemistry and immunofluorescence were applied to verify key genes and cell marker molecules in clinical samples. Immunohistochemistry, quantitative real-time PCR, and western blotting were performed to examine the expression of IL-1β and MMP9. Moreover, quantitative analysis and sorting of myeloid-derived suppressor cells (MDSCs) and CD8^+^ T cells were performed using flow cytometry.

**Results:**

Tumor responses in 23 patients with MSI-H/dMMR mCRC were evaluated by radiology. The objective response rate was 43.48%, and the disease control rate was 69.57%. ScRNA-seq analysis showed that, compared with the treatment-resistant group, the treatment-sensitive group accumulated more CD8^+^ T cells. Experiments with both clinical samples and mice indicated that infiltration of IL-1β-driven MDSCs and inactivation of CD8^+^ T cells contribute to anti-PD-1 resistance in MSI-H/dMMR CRC.

**Conclusions:**

CD8^+^ T cells and IL-1β were identified as the cell type and gene, respectively, with the highest correlation with anti-PD-1 resistance. Infiltration of IL-1β-driven MDSCs was a significant factor in anti-PD-1 resistance in CRC. IL-1β antagonists are expected to be developed as a new treatment for anti-PD-1 inhibitor resistance.

**Supplementary Information:**

The online version contains supplementary material available at 10.1186/s12916-023-02866-y.

## Background

Colorectal cancer (CRC) is one of the most common malignant digestive tract tumors and has the second highest mortality rate and the third highest incidence rate among all malignant tumors [1]. Although diagnosis and treatment strategies for CRC have improved rapidly in recent years, the prognosis is unfavorable for many CRC patients. Many patients are not diagnosed until they are already in advanced stages of disease, which is a significant obstacle to effective treatment.

The expression of proteins related to mismatch repair deficiency (dMMR) and microsatellite instability-high (MSI-H) status have been widely recognized as valuable for predicting the efficacy of immunotherapy in CRC patients. Compared to traditional cancer therapies, immunotherapy has improved the objective response rate (ORR) of MSI-H mCRC to some extent [2, 3]. However, the ORR of patients with MSI-H/dMMR mCRC with first-line anti-programmed cell death protein-1 (PD-1) monotherapy was only 43.8% [4]. Thus, more than half of patients do not benefit from immunotherapy or chemotherapy ± targeted therapy because of the MSI-H phenotype [5]. In view of this, this study aims to explore the mechanism of anti-PD-1 resistance in patients with MSI-H mCRC.

ScRNA-seq has contributed to a better understanding of the immune landscape in MSI-H patients, which in turn has offered novel insights into the mechanisms of immunotherapy. Previous studies have mainly focused on the mechanism of immune resistance in microsatellite stable (MSS) mCRC. Several studies have revealed the molecular mechanism of CD40 and CD73 antagonist therapy at the single-cell level, providing a possible theoretical basis for their combined treatment with immune checkpoint inhibitors for MSS mCRC [6–8]. However, the mechanism of PD-1 resistance in patients with MSI-H mCRC is still unclear. Therefore, comparing the immune microenvironment between anti-PD-1-resistant and anti-PD-1-sensitive groups by scRNA-seq could help elucidate the mechanisms underlying immunotherapy resistance in MSI-H mCRC patients.

In the present study, tissue samples were collected during colonoscopy from treatment-sensitive and -resistant groups of MSI-H mCRC treated with a PD-1 blocker (tislelizumab). Next, we comprehensively analyzed the cell subtypes and key genes of the two groups using scRNA-seq. Follow-up experiments were performed using clinical samples and mouse models to verify the potential mechanism of anti-PD-1 resistance induced by the key cell types or genes indicated by scRNA-seq.

## Methods

### Sample collection

23 MSI-H/dMMR mCRC patients were treated with anti-PD-1 monotherapy at Yunnan Cancer Hospital (The Third Affiliated Hospital of Kunming Medical University) between August 1, 2020, and May 31, 2022. A PD-1 blocker (200 mg, tislelizumab, Bei Gene Ltd., China) was injected intravenously on the 1st day of each 21-day cycle. Efficacy was evaluated radiologically after every third cycle of treatment. Patients were considered sensitive to anti-PD-1 treatment if they showed a complete response (CR) or partial response (PR), and patients were considered resistant to anti-PD-1 treatment if they had progressive disease (PD) or stable disease (SD). Fresh intestinal tumor tissues (2–4 mm) were taken during colonoscopy. Tissue samples from a total of 23 patients (10 sensitive and 13 resistant) were analyzed by immunohistochemistry (IHC) and immunofluorescence (IF). Six patients (3 PR and 3 PD) were randomly selected for scRNA-seq. All participants provided written informed consent before beginning the study. Additionally, the ethics committee of Yunnan Cancer Hospital approved all study protocols that involved human subjects according to the ethical principles described in the Helsinki Declaration. The inclusion criteria were as follows: primary colorectal adenocarcinoma confirmed by colonoscopy biopsy and distant metastasis confirmed by CT/MR; MSI-H/dMMR status confirmed by multiplex qPCR or IHC; undergoing first-line anti-PD-1 monotherapy. Patients were excluded if we were unable to obtain a sample through colonoscopy due to contraindications or we were unable to perform single-cell sequencing due to insufficient cell activity or quantity.

### Radiology and colonoscopy

Siemens (SOMATOM Definition AS +) 128 slice spiral CT was used for plain and enhanced scanning. Patients were fasting and had performed bowel preparation as directed. The scanning range covered the entire abdominal cavity. The scanning layer was 1.0 mm thick with an interval of 0.6 mm. Iohexol (300 mg/ml, 100 ml) was used as the contrast agent. The delay time of arterial phase scanning was 35–40 s, and that of venous phase scanning was 70–80 s. MRI was performed using a Philips Elision 3.0 T, and the scanning sequence included transverse T1WI_Tse, sagittal and coronal T2WI_Tse, high-resolution T2WI_Tse, diffusion-weighted imaging, and multiphase dynamic enhancement. Gadolinium diamine was used as the contrast agent. Colonoscopy was performed using the Olympus CV-290, and tissues were collected by doctors with at least five years of experience.

### Single-cell preparation

Fresh tumor tissues obtained from CRC patients were immediately transferred to MACS C-tubes (Miltenyi Biotec) with digestive enzymes. Then, digestion was performed by a gentleMACS Octo Dissociator (Miltenyi Biotec) (30 min at 37 °C). Single cells were processed by Chromium Controller (10X Genomics) according to the manufacturer's protocol. Briefly, cells were washed with 20 mL of RPMI 1640 (Gibco), filtered through a 70-μm nylon strainer (BD Falcon), collected by centrifugation (330 × g, 10 min, 4 °C), and resuspended in a basic solution containing 0.2% fetal bovine serum (FBS; Gibco).

### Single-cell capture and library preparation

A 10 × Chromium system (10 × Genomics) and library preparation by LC Sciences were utilized to run the single cells according to the recommended protocol for the Chromium Single Cell 30 Reagent Kit (v2 Chemistry). The Illumina HiSeq4000 was used for sequencing, and a 10 × Cell Ranger package (v1.2.0; 10 × Genomics) was used for the postprocessing and quality control of libraries.

### Quantitative analysis of the single-cell sequencing data

All single-cell sequencing data were analyzed by Cell Ranger V6.1.1. The results are shown in Additional file 1: Table S1. CRC samples from 3 resistant patients (named R1, R2, R3) and 3 sensitive patients (named S1, S2, S3) contained a total of 56,092 cells; the number of genes detected in each sample ranged from 17,564 to 18,093; the median number of cellular unique molecular identifiers ranged from 3,403 to 6,915; and the sequencing saturation ranged from 47 to 63%. These results indicate that, overall, the sequencing quality was sufficient for use in subsequent correlation analysis.

### Processing of CRC single-cell sequencing data

In total, this analysis included 56,092 cells from the sensitive group and resistant group. The Seurat package in R (version 4.0.5) was used for analysis and quality control [9]. Low-quality cells (*n* = 3,071) were removed if genes were detected only in < 3 cells or if there were < 200 total genes detected by gene-cell matrixes. Next, the global-scaling method LogNormalize was performed to normalize the gene expression values for the remaining 53,021 cells. Then, the FindVariableFeatures function combined with the vst method in R studio was employed to identify the most variable genes, which were used for dimensionality reduction. After principal component analysis, 2000 highly variable genes were identified. Jackstraw and ScoreJackStraw functions were applied to determine the most significant principal components. Finally, graph-based unsupervised clustering was conducted and visualized using a nonlinear t-distributed stochastic neighbor embedding (t-SNE) plot, defined by the FindNeighbors and FindClusters functions.

### Identification and characterization of cell subtypes

The identities of cell types were characterized using the SingleR (V1.6.1) package based on the Celldex database. The FindMarkers function in the R package Seurat was used to list the markers of each cell cluster with min.pct = 0.5, logfc.threshold = 1, min.diff.pct = 0.3, and *P* < 0.05. The markers used in this pipeline are listed in Additional file 2: Table S2.

To investigate the molecular mechanisms involved in each cell subtype, biological process (BP), cell composition (CC), and molecular function (MF) GO enrichment analysis and KEGG pathway analysis were performed using the R package clusterProfiler (version 3.14.3), with the threshold of significance set to adjusted (adj.) *P* < 0.05.

### Screening of differentially expressed genes (DEGs)

The first 2000 highly variable genes were screened using the FindVariableFeatures function combined with the vst method. The FindMarkers function in the R package Seurat was used not only to find the marker genes for different cell subtypes (with screening parameter thresholds of min.pct = 0.5, logfc.threshold = 1, min.diff.pct = 0.3, *P* < 0.05) but also to identify DEGs of each cell subtype between anti-PD-1-sensitive and anti-PD-1-resistant samples (with the threshold set to |avg_log_2_FC|≥ 0.5, *P* ≤ 0.05). The overlapping genes between pseudotime-related genes and PD-1 resistance-related DEGs were considered to be candidate key genes.

### Pseudotime analysis

To reveal differences in immune cells in the sensitive and resistant groups, Monocle software (version 2.20.0) [10] was used to analyze sample trajectories and explore the differentiation process. First, a more sophisticated method (dpFeature) was created based on the foundation of cluster and custom developmental marker genes of Monocle. The signature genes with a high degree of dispersion (q < 0.01) were identified among cell subtypes that were selected by dpFeature. Next, DDRTree was applied for dimensionality reduction and pseudotemporal alignment of cells along the trajectory, and finally, the trajectories were visualized as 2D t-SNE maps. Pseudotime-related genes were identified based on q < 0.05 of the abovementioned signature genes.

### Protein‒protein interaction analysis of candidate key genes

The Search Tool for the Retrieval of Interacting Genes (STRING; http://string.embl.de/) was used to perform protein‒protein interaction (PPI) analysis on candidate key genes. The STRING database can be used to assess the direct (physical) and indirect (functional) associations of proteins [11]. Cytoscape 3.6.1 was used to establish a network model of PPI analysis results. Based on the STRING online tool, the PPI network of the candidate key genes was constructed with medium confidence = 0.4. The top four genes were selected as the key genes in this study based on the connectivity (degree) of each node in the PPI network.

### Cell culture and transfection

Colorectal cancer cell line CT26 was selected for this study. The plasmid used for cell transfection was synthesized by Ono Company. Eighteen to twenty-four hours prior to lentivirus infection, 3 × 10^5^/well adherent cells were spread in 6-well plates. The number of cells transfected with lentivirus was approximately 6 × 10^5^/well. When cells adhered to the wells and were 70% confluent, the original culture medium was replaced by 2 ml fresh culture medium containing 8 μg/ml polyzoan and a proper amount of virus suspension. Cells were then incubated at 37 °C for 8 h, after which the virus-containing medium was replaced with fresh medium. If transfection was successful, fluorescent protein was visible after 48–72 h. If no fluorescence was observed, the infection protocol was repeated. Puromycin was added to screen for lentivirus overexpression for one month.

### Animal experiments

All animal experiments were approved by the Animal Ethics Committee of Kunming Medical University. In the process of experimental operation, we strictly follow the ARRIVE guidelines. Throughout the experiment, researchers did not know which group the animals taken out of the cage would be assigned to, animal managers and researchers doing the experiment did not know the assignment sequence, and researchers evaluating, testing or quantifying the results of the experiment did not know the means of intervention.

BALB/c mice (male, aged 6–8 weeks old, 20-30 g) were purchased from Beijing Sipeifu Biotechnology Co., Ltd. All mice were maintained in an SPF room in the animal-housing facilities at the Kunming Medical University with food and water provided at will. The experiment was carried out after 1 week of adaptive feeding. Based on the degree of freedom (E) of variance analysis proposed by Mead, we estimated the sample size of the experimental animals we need [12]. The total sample size of mice in this study was 25, and they were randomly divided into 5 groups with 5 mice in each group. All mice were randomly divided into 5 groups using a simple random sampling method, defined as OE-NC, OE-IL-1β, OE-IL-1β + Diacerein, OE-IL-1β + Nivolumab, and OE-IL-1β + Diacerein + Nivolumab groups, respectively. For the control group model (OE-NC group), 1 × 10^6^ empty vector stabilized cells in 100ul PBS were subcutaneously injected into the flank of mice. For the treatment model, 1 × 10^6^ over-expressed IL-1β stably transfected cells in 100ul PBS were subcutaneously injected into the same site of mice. When the tumor volume reached 40mm^3^ (i.e. the 7th day), the OE-IL-1β + Diacerein group began intraperitoneal (i.p.) injection of the IL-1β antagonist (Diacerein, 0.07 mg/kg), OE-IL-1β + Nivolumab group received i.p. injection of anti-PD-1 antibody (Nivolumab, 2 mg/kg); The OE-IL-1β + Diacerein + Nivolumab group was treated with a combination of diacerein and Nivolumab intraperitoneally. The mice in the OE-NC group and OE-IL-1β group were administered the same volume of PBS. Diacerein and Nivolumab were intraperitoneally injected every 3 days from the 7th day of tumor cell inoculation.

The weight of mice and the volume of tumor were measured in 0d, 7d, 10d, 12d, 14d and 16d in each group, respectively. Tumor volume was calculated as ½ (Length × Width2). On the 16th day after inoculation of tumor cells, all mice were sacrificed with an intraperitoneal injection of sodium pentobarbital (200 mg/kg). Determine mice death based on the disappearance of corneal reflex and the emission of pupils. Tumor tissues were stripped and weighed, and subsequent molecular biological experiment were performed. The selection of 16d as the experimental endpoint is based on the pre- experiment results. The selection of 16d as the experimental endpoint is based on the pre- experiment results. Throughout the entire experiment, the length of tumor should not exceed 20 mm, and the tumor weight should not exceed 10% of the mice weight [13].

### Quantitative real-time PCR

Total RNA was extracted from the cultured cell line CT26 or tissues using TRIzol reagent (Ambion) and reverse transcribed into cDNA by the SureScript first strand cDNA synthesis kit (Servicebio) according to the manufacturer’s instructions. 2xUniversal Blue SYBR Green qPCR Master Mix (Servicebio) and CFX96 sequence detection system (Bio-Rad, Hercules, CA, USA) were used for qPCR, and the following primers were used: IL-1β (human), forward: 5'- AATCTCCGACCACCACTACA-3' and reverse: 5'-GACAAATCGCTTTTCCATCT-3'; MMP9 (human), forward: 5'-ATGAGCCTCTGGCAGCCCCTGGTCC-3' and reverse: 5'- GGACCAGGGGCTGCCAGAGGCTCAT-3'; GAPDH (human), forward: 5'-CCCATCACCATCTTCCAGG-3' and reverse: 5'-CATCACGCCACAGTTTCCC-3'; IL-1β (mouse), forward: 5'- CCTATGTCTTGCCCGTGG-3' and reverse: 5'- GTGGGTGTGCCGTCTTTC-3'; MMP9 (mouse), forward: 5'-GTGTGTTCCCGTTCATCTTT-3' and reverse: 5'- GCCGTCTATGTCGTCTTTAT-3'; GAPDH (mouse), forward: 5 '- CCTTCCGTGTTCCTACCCC-3' and reverse: 5'-GCCCAAGATGCCCTTCAGT-3'. GAPDH was used as a standardized endogenous control, and 2^−△△CT^ was used to calculate the relative mRNA expression.

### Western blotting

Protein samples were isolated from tissues or cells using RIPA lysis buffer (Servicebio, Wuhan, China) containing 1% protease and phosphatase inhibitors (PMSF; Servicebio). A BCA protein assay kit (Biyuntian Biotechnology) was used for protein quantification. Sodium dodecyl sulfate‒polyacrylamide gel electrophoresis was applied to separate proteins of different molecular weights, and proteins were transferred to a polyvinylidene fluoride (Servicebio) membrane. The membrane was blocked with 5% skim milk for 90 min at room temperature and subsequently incubated with primary antibodies (Proteintech; IL-1β 1:1000; MMP9 1:1000; β-actin 1:25,000) at 4 °C overnight, followed by incubation with HRP-conjugated secondary antibodies for 2 h at room temperature.

### Immunohistochemistry and immunofluorescence

Paraffin sections of tissues were deparaffinized and rehydrated. Then, sodium citrate buffer was used to extract the antigens to be detected, and antigen retrieval was completed by heat induction. Sectioned tissues were incubated with 3% H_2_O_2_ for 15 min to block endogenous peroxidase activity (IF proceeded without this step) and then blocked with PBS containing 5% fetal bovine serum for 30 min. Tissues were incubated with primary antibodies overnight at 4 degrees C, followed by incubation in the dark with the conjugated secondary antibodies at room temperature for another 2 h. DAB was used as nuclear markers for IHC. DAPI (EX:330-380 nm, Em:420 nm) was used to stain the cell nuclei (blue), Alexa Fluor 488 (EX:495 nm, Em:519 nm) was used to stain CD11b (green), Alexa Fluor 555(EX:555 nm, Em:565 nm) was used to stain CD14, CD15 and CD8 (red), Alexa Fluor 594 (EX:590 nm, Em:617 nm) was used to stain CD33 (orange).

### Flow cytometry

Polymorphonuclear (PMN)-MDSCs/monocytic (M)-MDSCs were stained with CD11b-FITC (Biolegend, 101,205, USA), Ly-6G-PE (Biolegend, 127,607, USA), and Ly-6C-APC (Biolegend, 128,016, USA), and CD8^+^ T cells were stained with CD3-FITC (Biolegend, 100,203, USA) and CD8-PE (Biolegend, 100,707, USA) according to the manufacturer’s instructions. Samples were run on a Guava easyCyte 8HT flow cytometer (Millipore). Forward and side scatter gating were performed using FlowJo_V10.

### Statistical analysis

Bioinformatics analyses were performed using R software. The varElect online tool was applied to analyze the correlation between genes in the PPI network and CRC. A high score indicated a strong correlation, with a significance threshold of *P* < 0.05 unless otherwise stated. All data are presented as the mean ± standard error (SE) of independent experiments. Two-tailed one-way analysis of variance (ANOVA) with multiple comparison post hoc analysis was used, and *P* values < 0.05 (*), *P* < 0.01 (**), *P* < 0.001 (***), and *P* < 0.0001 (****) are indicated as significant. Statistical analysis was performed using GraphPad Prism 9.0.

## Results

### Efficacy evaluation for MSI-H/dMMR mCRC after anti-PD-1 monotherapy

**Fig. 1:**
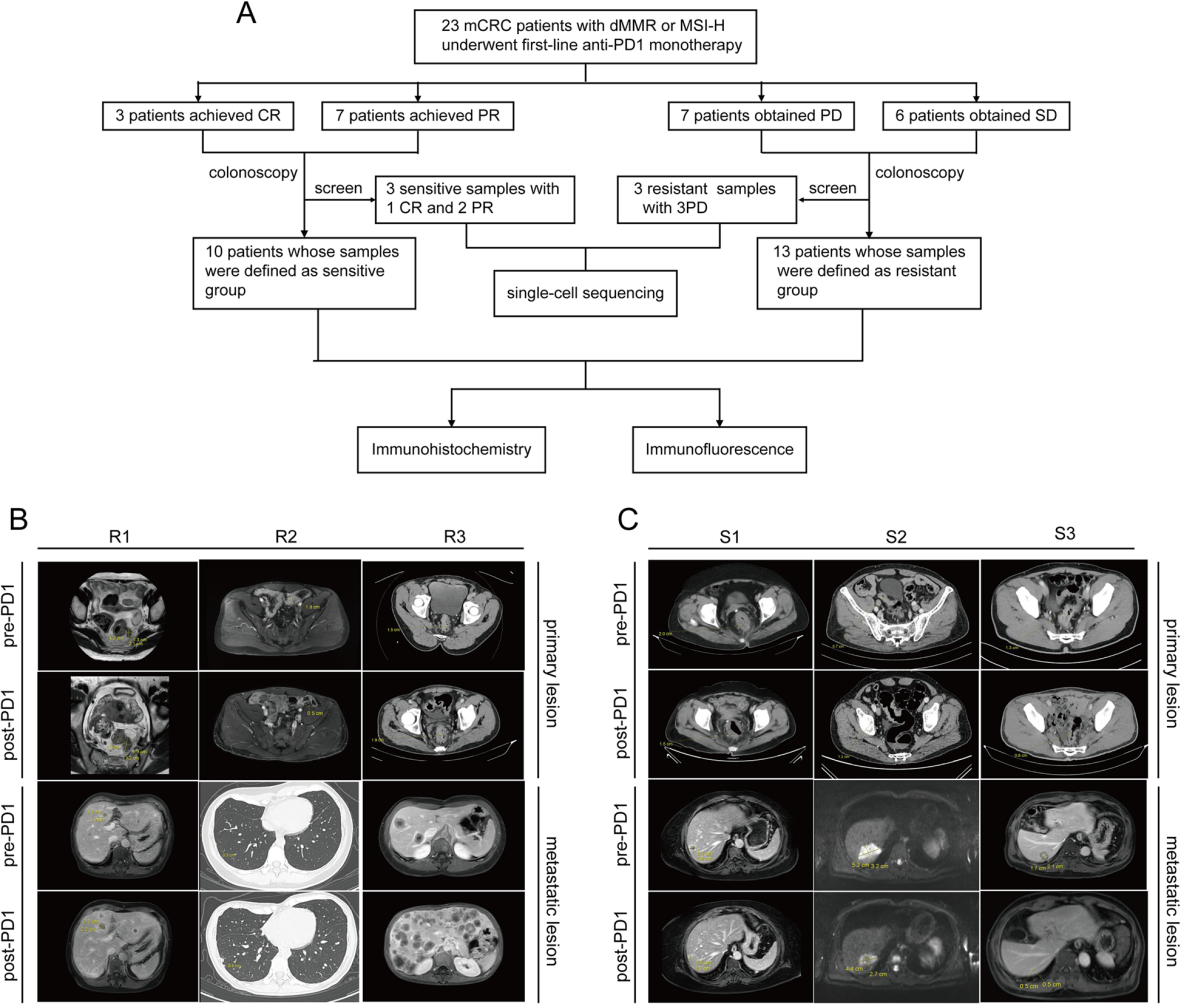
Efficacy evaluation of MSI-H mCRC after first-line PD-1 monotherapy. **A** Flow chart for screening 23 patients receiving first-line PD-1 monotherapy. Images of primary and metastatic lesions pre- and post-immunotherapy are shown for (**B**) three resistant patients and (**C**) three sensitive patients

A total of 23 MSI-H/dMMR mCRC patients were treated with first-line anti-PD-1 monotherapy between August 1, 2020, and May 31, 2022. Treatment response was evaluated by radiological examination after every 3 cycles of PD-1 inhibitor. PR was recorded for seven patients, CR was recorded for three patients, SD was recorded for six patients, and PD was recorded for seven patients. The ORR was 43.48% (10/23), and the disease control rate (DCR) was 69.57% (16/23) (Fig. 1A). The radiological findings for primary and metastatic lesions in six patients pre- and post-immunotherapy can be seen in Fig. 1B and C. Figure 1B shows the changes in primary and metastatic lesions in 3 patients in the resistance group (R1, R2 and R3) after anti-PD-1 treatment. The length of the primary lesions of R1 increased from 2.1 cm to 3.2 cm, while that of the metastatic lesions increased from 1.6 cm to 2.7 cm; the primary and metastatic lesions of R2 increased from 0.5 cm to 1.9 cm and from 0.3 cm to 0.8 cm, respectively; the length of primary lesions of R3 increased from 1.5 cm to 1.9 cm, and the number of metastatic lesions increased from 3 to more than 20. The responses of R1, R2 and R3 were evaluated as PD. Similarly, Fig. 1C shows the changes in primary and metastatic lesions in 3 patients in the sensitive group (S1, S2 and S3) after anti-PD-1 monotherapy. The length of the primary tumor of S1 decreased from 2.0 cm to 1.5 cm and that of the metastatic tumor decreased from 2.1 cm to 1.2 cm; the lengths of the primary and metastatic lesions of S2 decreased from 1.2 cm to 0.7 cm and from 5.3 cm to 4.4 cm, respectively; and the lengths of the primary and metastatic lesions of S3 decreased from 1.3 cm to 0.8 cm and from 2.1 cm to 0.5 cm, respectively. The responses of S1, S2 and S3 were evaluated as PR.

### Identification of 23 cell clusters based on single-cell sequencing data from CRC samples

**Fig. 2:**
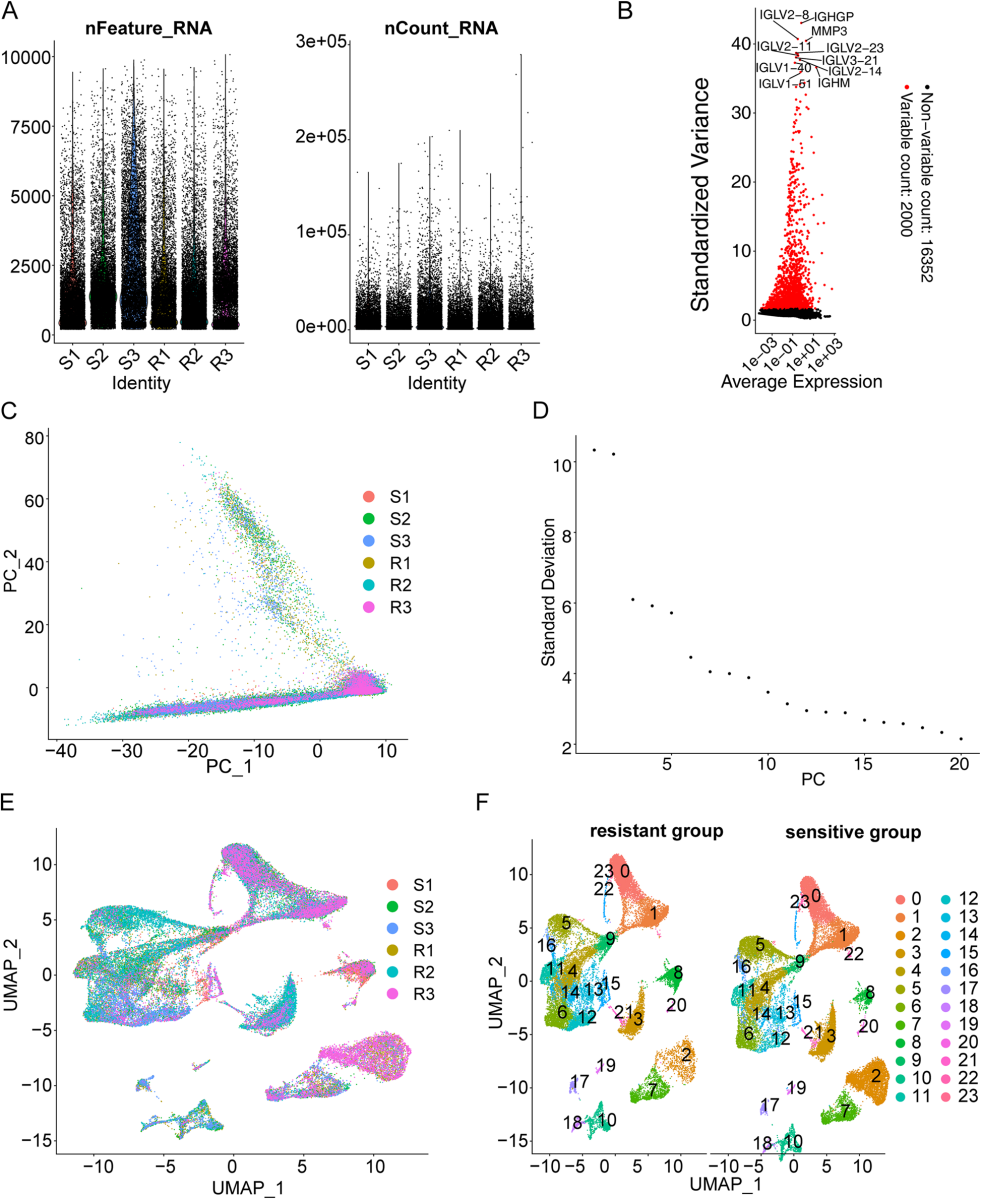
Cell clusters in six CRC samples were identified by scRNA sequencing. **A** Number of cells in different samples. **B** Display of the top 2000 variant genes. **C** PCA of the top 2000 variant genes. **D** Linear dimensionality reduction analysis of principal components. **E** Analysis of cell distributions by nonlinear dimensionality reduction combined with the RunMap function. **F** Analysis of cell cluster distributions in the sensitive and resistant groups through t-SNE

A total of six patients (three PR and three PD) were randomly selected for scRNA-seq. 10 × Genomics scRNA sequencing datasets were obtained from fresh CRC tissues obtained from three resistant (named R1, R2, R3) and three sensitive (named S1, S2, S3) patients. After removing 3071 low-quality cells, a total of 53,021 cells were used in the final analysis (Fig. 2A); specifically, there were 7679 cells from R1, 10,797 cells from R2, 9020 from R3, 7880 cells from S1, 9369 cells from S2, and 8276 cells from S3. Figure 2B shows the top 2000 highly variant genes, with the ten most variable genes labeled. PCA of the 2000 highly variable genes demonstrated no significant separation in CRC cells between resistant and sensitive groups (Fig. 2C). Twenty principal components were selected based on linear dimensionality reduction analysis (Fig. 2D). Nonlinear dimensionality reduction of the data according to the dimension value 20 combined with the RunMap function revealed a more uniform distribution of cells in each sample (Fig. 2E). Subsequently, these cells were classified into 23 cell clusters based on gene expression levels by t-SNE, and the distribution of these cellular taxa was found to be nearly identical across the sensitive and resistant groups (Fig. 2F).

### Characterization of the nine cell subtypes

**Fig. 3:**
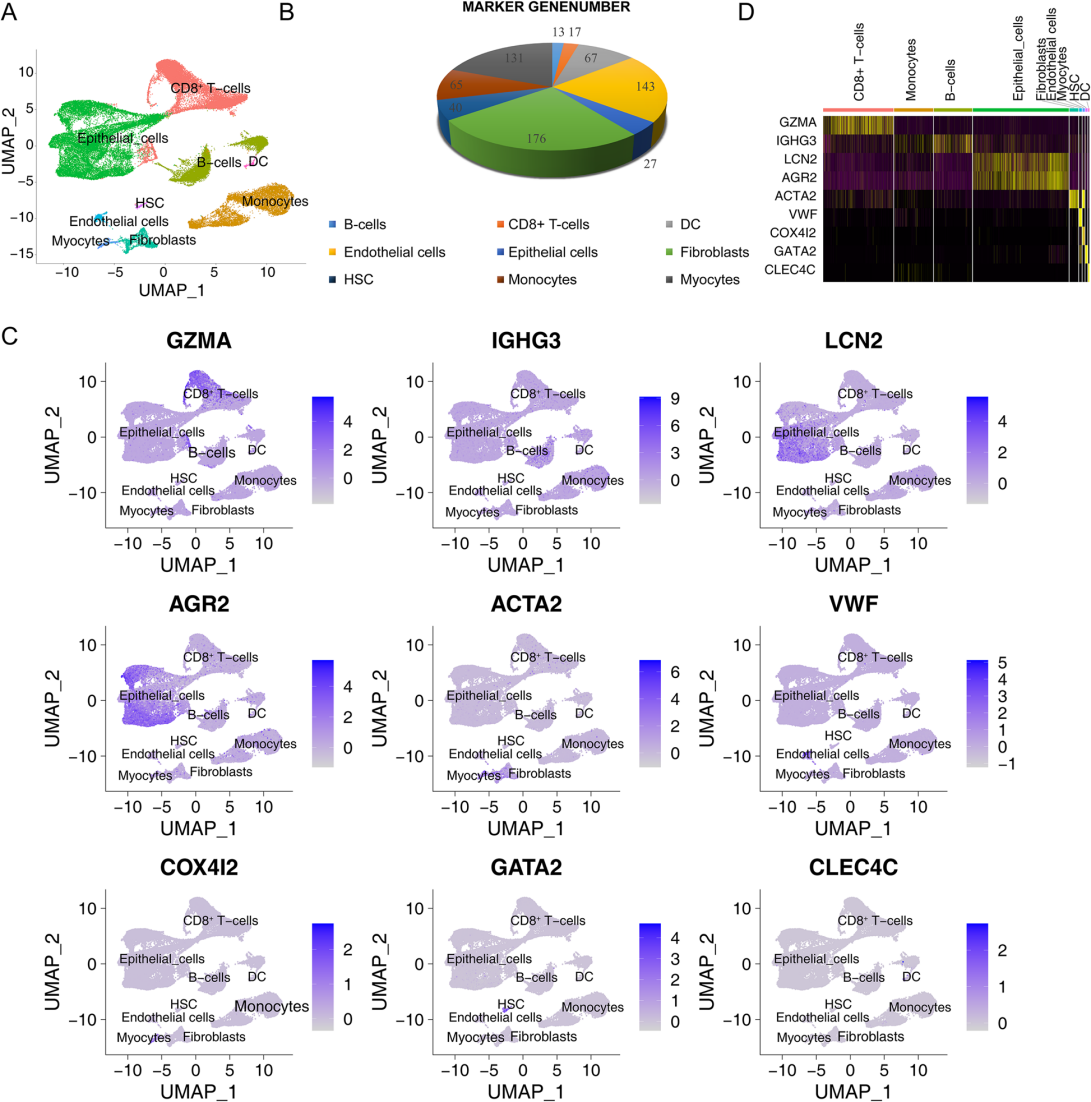
Annotation of cell subtypes and functional enrichment analysis of marker genes. **A** Annotation of cell subtypes according to the R package SingleR and celldex. **B** Number of marker genes for each cell subtype. **C** Scatter plot showing the top gene marker for each subtype. **D** Heatmap showing the top gene maker for each subtype

Using the R packages SingleR and celldex, 23 cell clusters were annotated into nine cell subtypes: CD8 + T cells, epithelial cells, B cells, dendritic cells (DCs), hematopoietic stem cells, monocytes, fibroblasts, myocytes, and endothelial cells (Fig. 3A). Combined with Fig. 2F, the aggregation of CD8^+^ T cells and monocytes in the sensitive group was significantly higher than that in the resistant group. The number of cells of each cellular subtype in each sample is shown in Additional file 3: Table S3. A total of 679 marker genes were obtained for the nine cell subtypes (Additional file 2: Table S2): there were 176 markers for fibroblasts, 143 markers for endothelial cells, 131 markers for myocytes, 67 markers for DCs, 65 markers for monocytes, 40 markers for hematopoietic stem cells, 27 markers for epithelial cells, 17 markers for CD8^+^ T cells, and 13 markers for B cells (Fig. 3B). The top gene for each cell subtype is shown in Fig. 3C. The heatmap of these genes illustrated that each cluster displayed distinct gene expression features (Fig. 3D).

GO analysis showed that these marker genes were mainly enriched in immune-related biological processes such as differentiation of lymphocytes and monocytes and positive regulation of leukocytes. Moreover, they were also significantly associated with “cytokine-mediated signaling pathway” (Additional file 4: Figure S1A-C). KEGG enrichment analysis (Additional file 4: Figure S1D) indicated that the marker genes were significantly correlated with immune/inflammatory responses (e.g.“cytokine − cytokine receptor interaction”, “Fc epsilon RI signaling pathway”, “AGE − RAGE signaling pathway”, “cell adhesion molecules”). Cancer-related pathways, including “PI3K-Akt signaling pathway” and “proteoglycans in cancer”, were also significantly enriched.

### Simulation of cell cluster developmental trajectories and screening of key genes

**Fig. 4:**
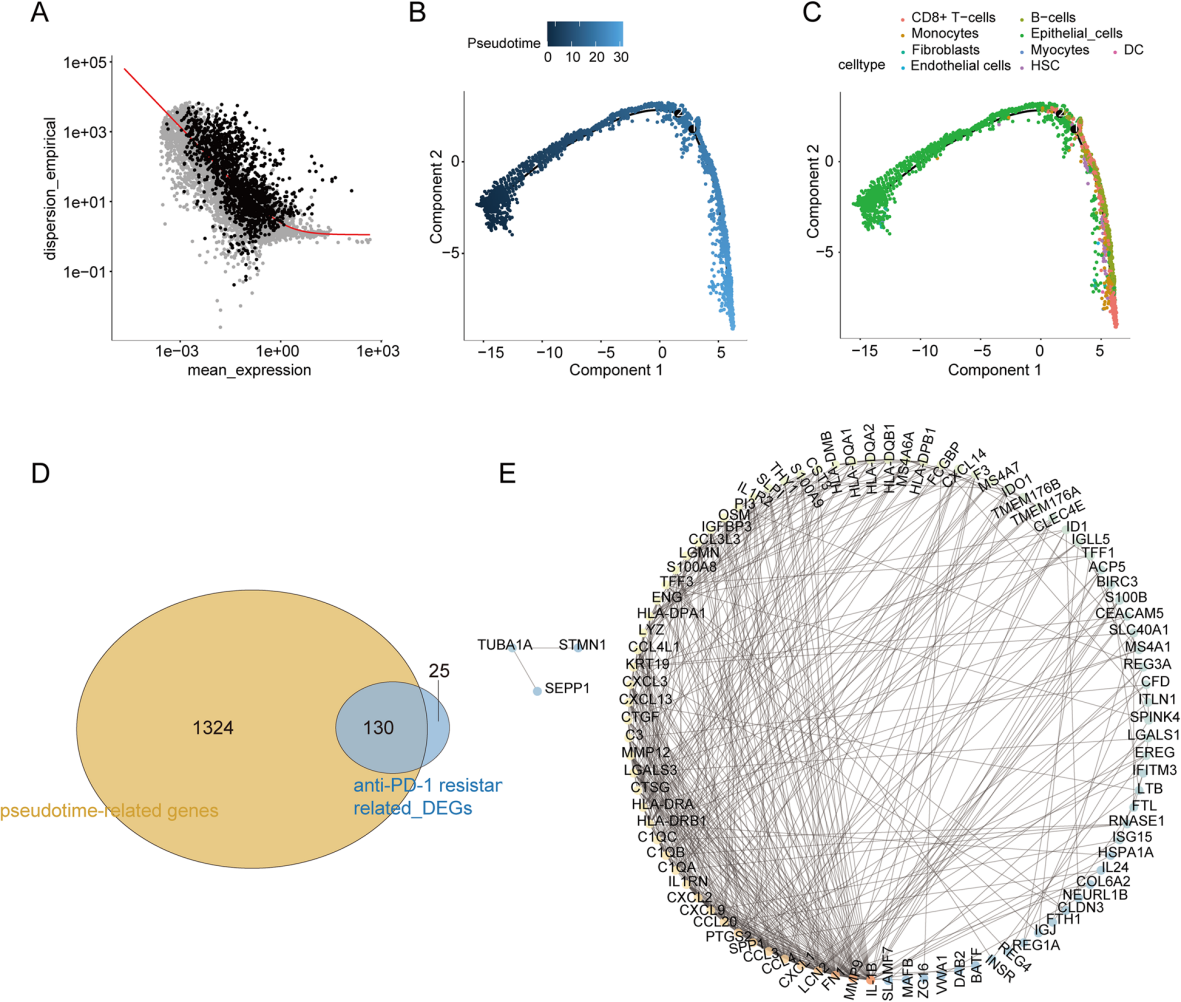
Construction of cell clusters developmental trajectories and identification of key genes. **A** Relative dispersion of feature genes. **B** Construction of the tree structure of the differentiation trajectory spectrum. **C** Temporal evolutionary trend of cell subtypes. **D** Identification of common genes through overlap analysis. **E** A PPI network based on 130 common genes

Developmental trajectories were simulated for the nine cell subtypes, and 1623 feature genes were found to be differentially expressed among the cell subpopulations (Additional file 5: Table S4). Figure 4A illustrates the relatively high dispersion of these feature genes. Individual cells were then classified by these feature genes using the R package Monocle, and a tree structure of the entire spectrum of differentiation trajectories was constructed (Fig. 4B). From a cell typing perspective, overall, the proposed temporal evolutionary trend of the cell subtypes was a gradual transition from epithelial cells to intermediate cells (e.g., fibroblasts, hematopoietic stem cells, monocytes, B cells) and eventually to CD8^+^ T cells (Fig. 4C). A total of 1454 pseudotime-related genes were identified from the 1623 feature genes (*P* < 0.05; Additional file 6: Table S5). KEGG analysis (Additional file 7: Figure S2A) revealed that these genes were involved in “cytokine‒cytokine receptor interaction”, “viral protein interaction with cytokine and cytokine receptor”, and “cell cycle”; they were also inextricably linked to the tumor-associated pathways “NF-kappa B signaling pathway”, “p53 signaling pathway”, and “PPAR signaling pathway”. GO-BP analysis revealed that these pseudotime-related genes were related to the immune response, inflammatory response, immune cell differentiation, proliferation, migration, and chemotaxis (Additional file 7: Figure S2B). Moreover, these genes also performed molecular functions such as “antigen binding”, “immunoglobulin receptor binding”, and “extracellular matrix structural constituent” (Additional file 7: Figure S2C) in cellular fractions such as “immunoglobulin complex”, “external side of plasma membrane”, and “immunoglobulin complex, circulating” (Additional file 7: Figure S2D).

To observe differences in gene expression for each cell subtype between the sensitive and resistant groups, we performed differential analysis using the FindMarkers function in the Seurat package. A total of 155 DEGs were obtained; 97 were upregulated, and 98 were downregulated (Additional file 8: Table S6). Functional enrichment analysis indicated that these genes were related to immune and inflammatory responses. DEGs in the CD8^+^ T-cell subtype were mainly involved in the IFN-related response and oxygen transport, while DEGs in the DC and fibroblast subtypes were associated with the chemokine-related response and neutrophil migration (Additional file 9: Figure S3A and B). Enrichment results for GO-CC and GO-MF are presented in Additional file 9: Figure S3B and C. KEGG enrichment analysis showed that DEGs in the B-cell and monocyte subtypes were involved in similar pathways and were enriched in antigen processing and presentation; DEGs in the DC and fibroblast subtypes were involved in cancer-related pathways, including chemokine and IL-17 signaling (Additional file 9: Figure S3D).

Through overlap analysis, 130 common genes were identified among 1454 pseudotime-related genes and 155 (deduplicated) PD-1 resistance-related genes (Fig. 4D; Additional file 10: Table S7). A PPI network containing 109 nodes and 435 edges was drawn based on the 130 common genes using the STRING database (Fig. 4E). VarElect analysis of the 130 common genes identified the two genes with the highest connectivity in the PPI network as IL-1β (score = 17.72) and MMP9 (score = 13.45), indicating that these two genes are most closely associated with CRC (Additional file 11: Table S8; Additional file 12: Figure S4). KEGG pathway enrichment analysis of these two key genes showed that they are involved in cytokine‒cytokine receptor interaction (IL-1β) and the MAPK and PI3K-Akt signaling pathways (IL-1β and MMP9). The specific signaling pathway maps are shown in Additional file 12: Figure S5.

### Relationship between IL-1β and MDSCs or CD8^+^ T cells in CRC patients

**Fig. 5:**
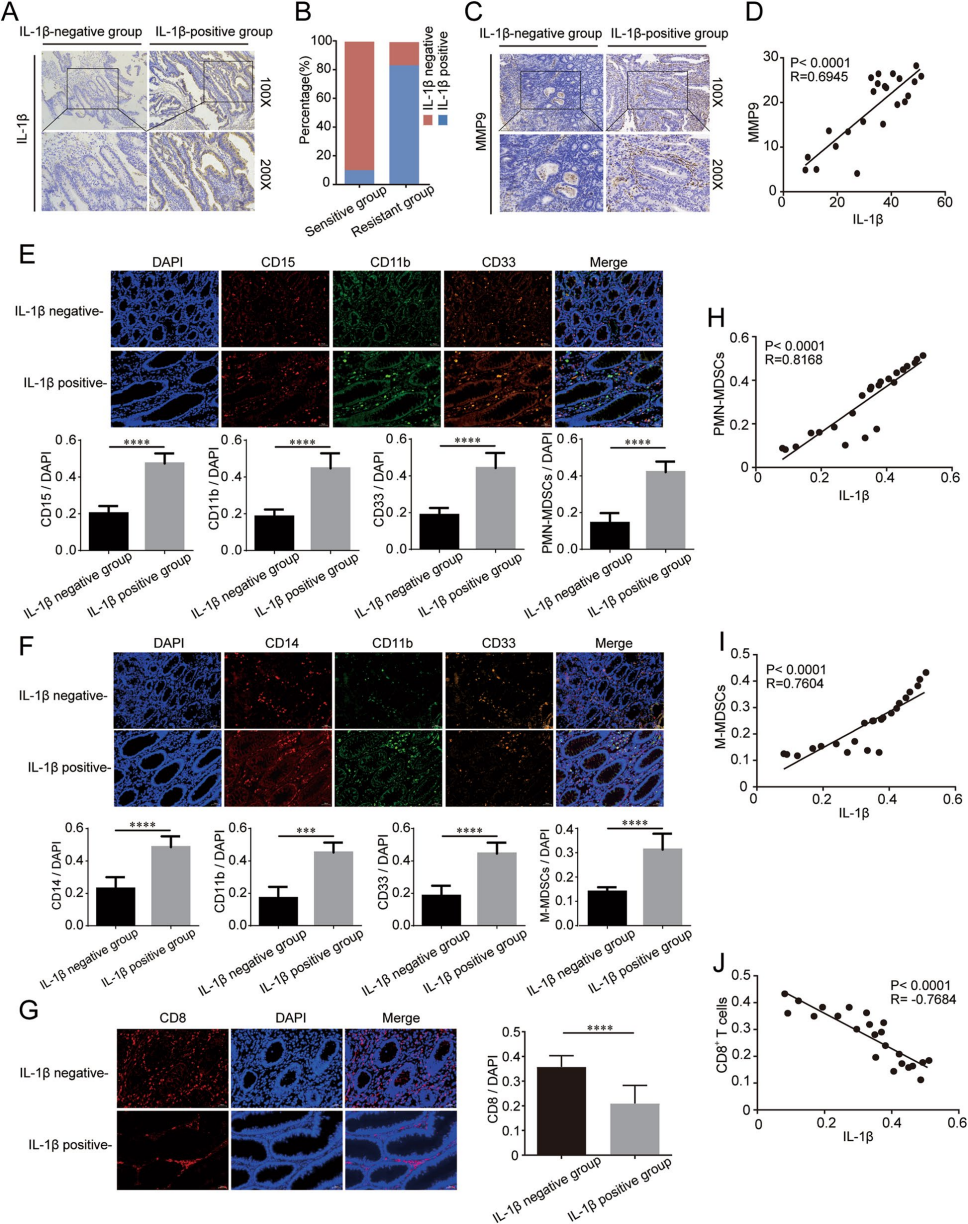
Relationship between IL-1β and MDSCs or CD8^+^ T cells in MSI-H/dMMR CRC patients. **A** IHC staining for IL-1β in CRC tissues. **B** Proportion of IL-1β-positive and IL-1β-negative tissues in the sensitive and resistant groups. **C** IHC staining for MMP9 in IL-1β-positive and -negative groups. **D** Correlation between MMP9 and IL-1β according to IHC scores. IF examination and quantification of (**E**) PMN-MDSCs, (**F**) M-MDSCs, and (**G**) CD8^+^ T cells in the IL-1β-negative and -positive groups. Pearson correlation analysis of IF scores between IL-1β and (**H**) PMN-MDSCs, (**I**) M-MDSCs, and (**J**) CD8.^+^ T cells. ****P < 0.0001

IL-1β and MMP9 were identified as the top two genes with the highest correlation with anti-PD-1 resistance through scRNA-seq. We used IHC to detect the expression of IL-1β and MMP9 in 10 sensitive and 13 resistant tumor tissues from MSI-H/dMMR patients. The IHC grade of IL-1β in IL-1β-positive tissues was significantly higher than that in IL-1β-negative tumor tissues (*P* < 0.001; Fig. 5A). Next, all 23 patients with were categorized into IL-1β-negative or IL-1β-positive groups based on the IHC grade of IL-1β. Of the 13 tissues from resistant patients, eleven tissues had high expression of IL-1β; of the 10 tissues from sensitive patients, only one tissue had high expression (Fig. 5B). A similar expression trend was observed for another key gene, MMP9 (*P* < 0.0001; Fig. 5C). MMP9 expression was positively correlated with IL-1β expression (*R* = 0.6945, *P* < 0.0001; Fig. 5D).

MDSCs are heterogeneous cells derived from bone marrow that lead to the inactivation of CD8^+^ T cells and immune resistance. Multiple studies have shown that IL-1β might play crucial roles in the aggregation and differentiation of MDSCs. We further examined the relationship between IL-1β expression and MDSCs using IF. The fluorescence intensity of makers in PMN-MDSCs and M-MDSCs in the tissues of IL-1β-positive patients was significantly higher than that in IL-1β-negative patients (*P* < 0.0001; Fig. 5E-F). CD8^+^ T cells were identified as one of the most significantly different cell clusters between the sensitive group and resistant group by scRNA-seq. CD8^+^ T cells in the tumor microenvironment (TME) are essential for the antitumor effects of immunotherapy. We used IF to detect the expression of CD8^+^ T cell marker in tissues and assessed the correlation between IL-1β and CD8^+^ T cells. The fluorescence intensity of CD8 in IL-1β positive tissue was significantly lower than that in IL-1β negative tissue (*P* < 0.0001; Fig. 5G). There was a significant correlation between IL-1β and PMN-MDSCs (*R* = 0.8168, *P* < 0.0001; Fig. 5H), M-MDSCs (*R* = 0.7604, *P* < 0.0001; Fig. 5I) or CD8^+^ T cells (*R* = 0.7684, *P* < 0.0001; Fig. 5J).

### Combining an IL-1β antagonist with an anti-PD-1 inhibitor overcame anti-PD-1 resistance in a xenograft model

**Fig. 6:**
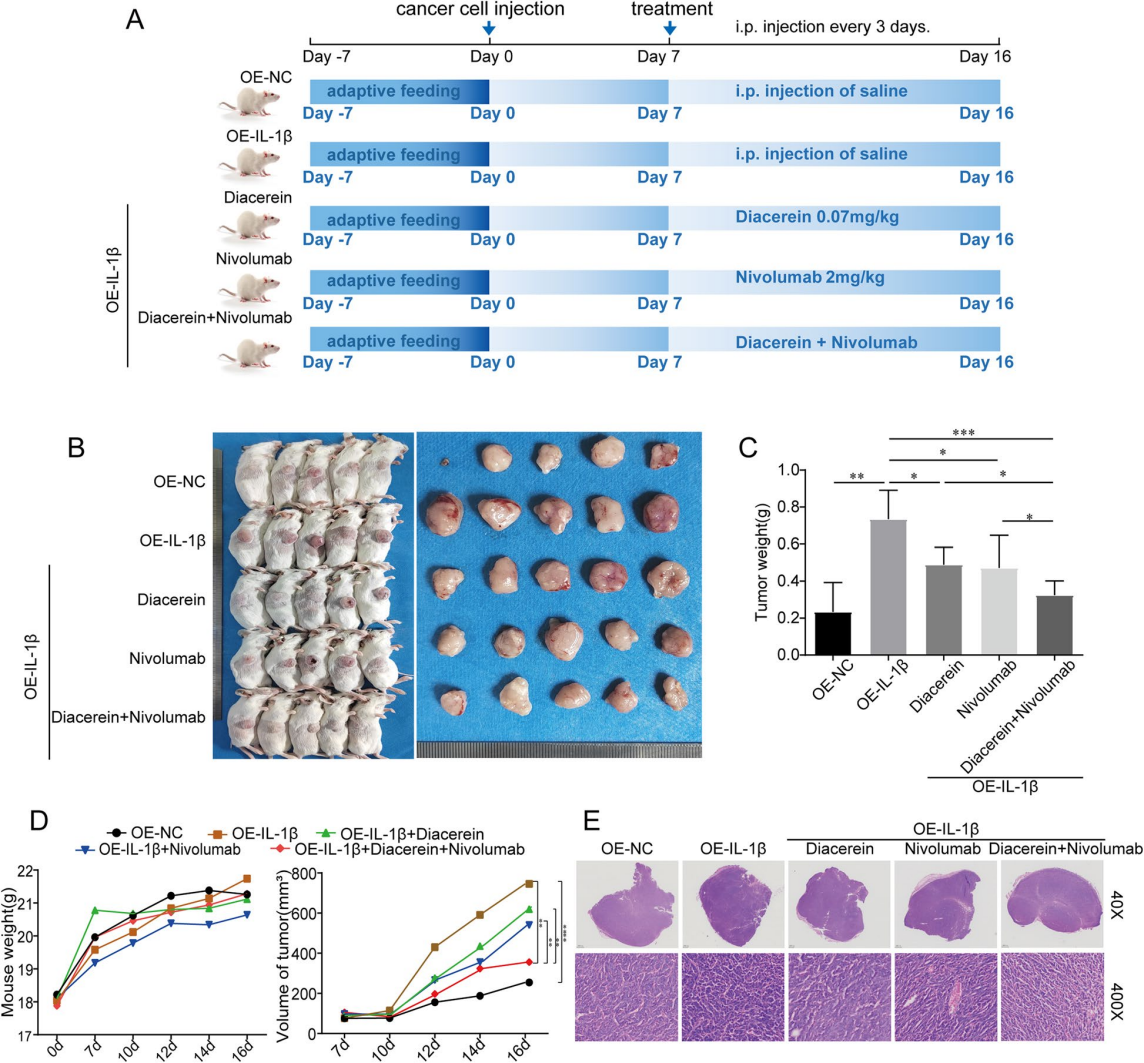
IL-1β antagonist and PD-1 inhibitor have a synergistic antitumor effect. **A** Diagram of the experimental timeline. **B** Xenograft tumors of BALB/c mice 16 days after inoculation with CT26 cells containing an empty vector or IL-1β overexpression vector (*n* = 5). **C** Tumor weight (*n* = 5). **D** Average body weight and average tumor volume of BALB/c mice in each treatment group (*n* = 5). **E** Hematoxylin–eosin staining of tumors from inoculated BALB/c mice (*n* = 5). *P < 0.05; **P < 0.01; ***P < 0.001; ****P < 0.0001

To further demonstrate the influence of IL-1β on PD-1 resistance in colorectal cancer, an in vivo xenograft model was used. The human IL-1β gene was stably transfected into CT26 cell lines to increase the expression of IL-1β, as verified by qPCR and western blotting (Additional file 12: Figure S6). IL-1β-overexpressing or untransfected control CT26 cells were then subcutaneously injected into male BALB/c mice (*n* = 5 each group; Fig. 6A and B). After 7 days of tumor cell inoculation, progressive tumor growth was observed. As shown in Fig. 6C-D, IL-1β upregulation resulted in a substantial increase in tumor volume, and there was no significant difference in mouse weight among the five groups. We treated the three groups with diacerein, nivolumab or both and observed the growth of tumors to determine whether the IL-1β antagonist cooperates with the immune agent to inhibit the growth of tumors. IL-1β-induced elevation of CRC tumor volume (745.74 ± 188.34) and weight (0.73 ± 0.16) was greatly attenuated by diacerein (619.59 ± 127.03, 0.49 ± 0.09) or nivolumab (540.47 ± 90.92, 0.49 ± 0.10, *P* < 0.05), and treatment with both drugs led to the greatest attenuation of tumor growth (355.49 ± 39.89, 0.32 ± 0.08, *P* < 0.001). Diacerein and nivolumab showed marked synergistic effects. Tumor sections from the inoculated mice are shown in Fig. 6E.

### IL-1β antagonism attenuates resistance to PD-1 by regulating MDSCs and CD8^+^ T cells

**Fig. 7:**
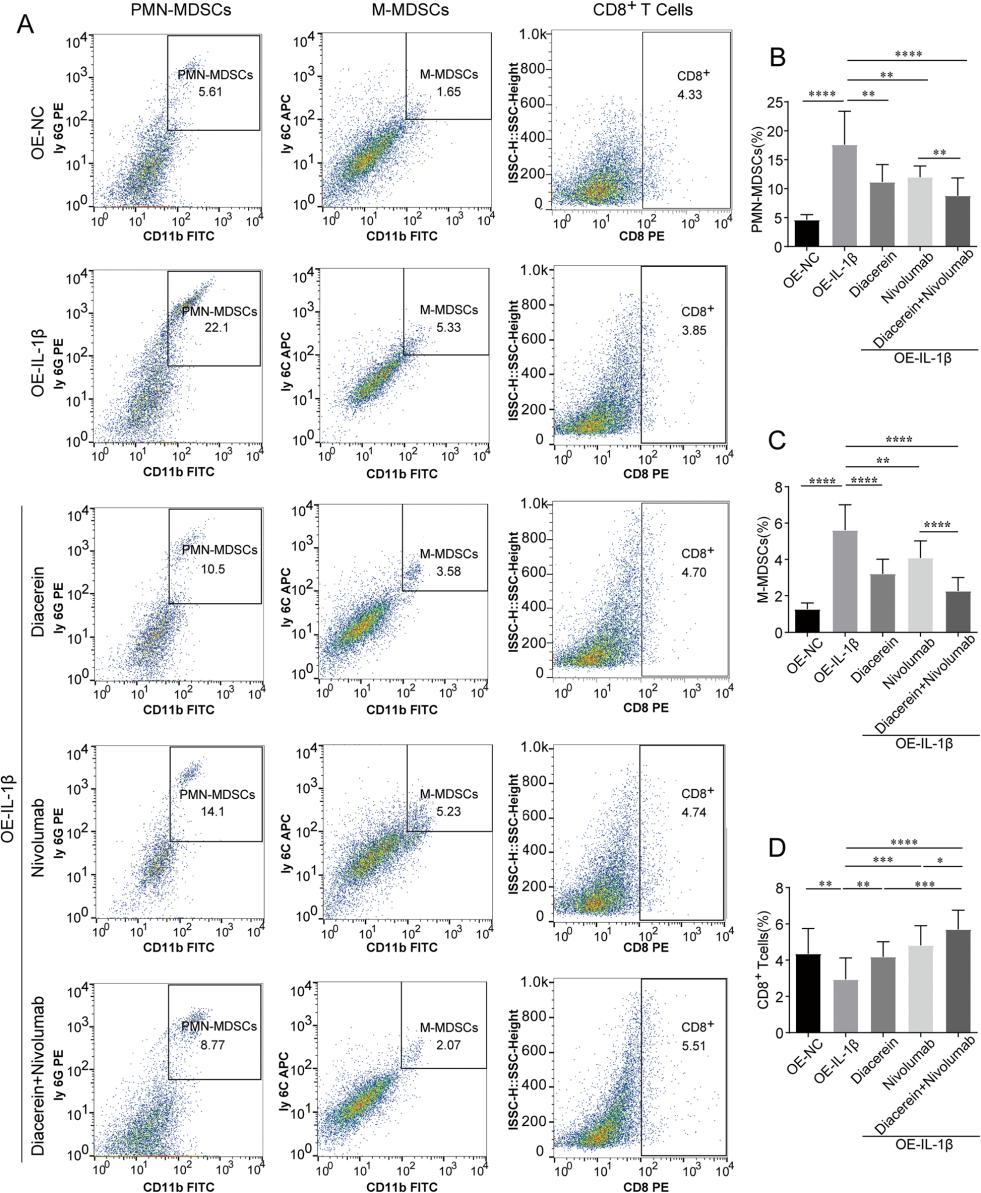
IL-1β stimulates the aggregation of MDSCs and inhibits the proliferation of CD8^+^ T cells in tumors. **A** Flow cytometry was performed to detect PMN-MDSCs, M-MDSCs, and CD8 + T cells (*n* = 5). Quantitative statistical diagram for expression of (**B**) PMN-MDSCs, (**C**) M-MDSCs, (**D**) CD8.^+^ T cells in each group (*n* = 5). *P < 0.05; **P < 0.01; ***P < 0.001; ****P < 0.0001

To examine whether the effectiveness of diacerein treatment against IL-1β-induced nivolumab resistance was mediated by MDSCs, flow cytometry was performed using biomarkers for PMN-MDSCs, M-MDSCs, and CD8^+^ T cells. As shown in Fig. 7, IL-1β resulted in a significant increase in the number of PMN-MDSCs (17.62 ± 5.56 *vs.* 4.64 ± 0.84, *P* < 0.0001) and M-MDSCs (5.61 ± 1.35 *vs.* 1.26 ± 0.33, *P* < 0.0001) in the TME, decreased the proportion of CD8^+^ T cells (2.94 ± 1.17 *vs.* 4.35 ± 1.37, *P* < 0.01) in the tumor tissues of mice, and enhanced immunosuppression. Next, we further assessed whether diacerein and nivolumab showed similar synergistic effects in reversing the IL-1β-mediated effect in PMN-MDSCs, M-MDSCs and CD8^+^ T cells. Monotherapy with either diacerein or nivolumab suppressed the differentiation of PMN-MDSCs (11.15 ± 2.90 *vs.* 17.621 ± 5.561, 12.0 ± 1.88 *vs.* 17.621 ± 5.561, *P* < *0.01*) and M-MDSCs (3.21 ± 0.77 *vs.* 1.26 ± 0.33, 4.09 ± 0.90 *vs.* 1.26 ± 0.33, *P* < *0.01*) and increased the number of CD8^+^ T cells (4.16 ± 0.76 *vs.* 2.94 ± 1.17, 4.81 ± 1.07 *vs.* 2.94 ± 1.17, *P* < 0.01). Figure 7B and C suggest that compared with nivolumab alone, the combined treatment significantly reduced the number of PMN-MDSCs *(*8.79 ± 2.98 *vs.* 12.0 ± 1.89, *P* < 0.01) and M-MDSCs (2.26 ± 0.72 *vs.* 4.09 ± 0.90, *P* < 0.001). Figure 7D shows that the degree of CD8^+^ T cell aggregation in the monotherapy groups with nivolumab (4.81 ± 1.07 *vs.* 5.695 ± 0.90, *P* < 0.05) was substantially reduced compared to that in the combination therapy group.

### Correlation between IL-1β and MMP9 expression levels in a mouse xenograft model

**Fig. 8:**
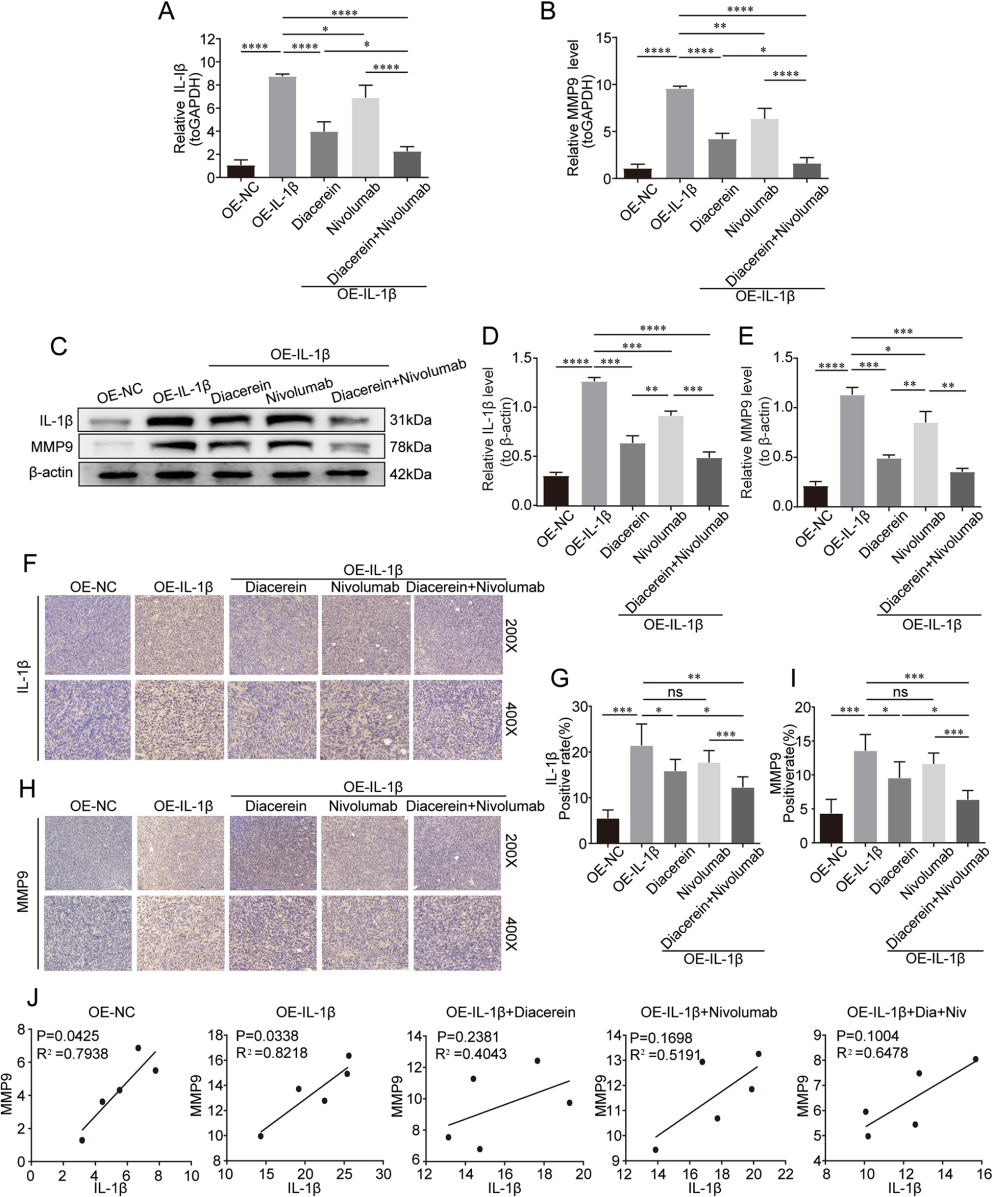
Correlation between IL-1β and MMP9 in the mouse xenograft model. qPCR of (**A**) IL-1β and (**B**) MMP9 mRNA expression in each treatment group (*n* = 3). **C** Western blotting analysis (*n* = 3). Grayscale analysis of (**D**) IL-1β and (**E**) MMP9 protein (*n* = 3). IHC staining and scores of (**F**, **G**) IL-1β and (H, I) MMP9 expression (*n* = 5). (J) Pearson correlation analysis of MMP9 and IL-1β expression (*n* = 5). *P < 0.05; **P < 0.01; ***P < 0.001; ****P < 0.0001

As one of the key genes related to resistance identified by single-cell sequencing, MMP9 is considered to have strong antivascular and immunosuppressive functions. However, the correlation between MMP9 and IL-1β remains unclear. MMP9 was detected in mouse CRC tissue using qPCR, western blotting, and IHC. As shown in Fig. 8, IL-1β treatment significantly increased MMP9 expression in mouse tumor tissues on the basis of the results of qPCR (9.57 ± 0.26 *vs.* 1.08 ± 0.44, *P* < 0.0001) and western blotting (1.13 ± 0.07 *vs.* 0.21 ± 0.04, *P* < 0.0001). Next, we assessed whether diacerein could cooperate with nivolumab to reduce the expression of MMP9. Monotherapy with either diacerein (4.21 ± 0.60 *vs*. 9.57 ± 0.26, *P* < 0.01) or nivolumab (6.36 ± 1.10 *vs.* 9.57 ± 0.26, *P* < 0.01) attenuated the IL-1β-induced upregulation of MMP9 through qPCR analysis. Combination therapy reduced MMP9 expression more potently than monotherapy with diacerein (1.62 ± 0.59 *vs.* 4.21 ± 0.60, *P* < 0.0001) or nivolumab (1.62 ± 0.59 *vs.* 6.36 ± 1.10, *P* < 0.0001). The expression of MMP9 in tumor tissue was almost positively correlated with that of IL-1β (the value of R2 is shown in Fig. 8I).

## Discussion

Compared with traditional chemotherapy or targeted therapy, immunotherapy has a unique molecular mechanism by which it shrinks tumors. It is generally considered that immune checkpoint inhibitors enhance the body’s general immunity by inducing T-cell activation and “releasing the immune brake” in the TME [14, 15]. In a previous study from our center, 29 patients with MSI-H/dMMR locally advanced colorectal cancer received neoadjuvant immunotherapy with a single-agent PD-1 inhibitor, and the ORR was 100% (29/29), consistent with the results of the NICHE study [16, 17]. In the present study, 23 patients with MSI-H/dMMR mCRC received first-line PD-1 inhibitor treatment, but the ORR was only 43.5% (10/23). These divergent responses of MSI-H mCRC to anti-PD-1 monotherapy are likely caused by differences in TME composition [18–20].

In view of this, through scRNA-seq, we defined various cellular subtypes, screened key genes, and revealed the TME signaling pathways that differ between the anti-PD-1 treatment-sensitive and -resistant groups. Together, the results suggested that the proportions of CD8^+^ T-cell subsets in the sensitive group were significantly higher than those in the resistant group [21–24]. Multiple CD8^+^ T-cell subsets in the TME may affect the response rate of new therapies targeting the immune system [21, 22]. MDSCs are heterogeneous cells derived from bone marrow that promote angiogenesis and immunosuppression [25]. Recent studies have shown that decreasing the aggregation of MDSCs in the TME can significantly increase the infiltration of CD8^+^ T cells and enhance the antitumor effects of PD-1 inhibition [26, 27]. The immunosuppressive effect of MDSCs might be related to the secretion of various cytokines, such as inducible nitric oxide synthase, arginase 1, interleukin-6, and transforming growth factor-β [28].

In this study, IL-1β ranked first in terms of connectivity in the overlap of pseudotime-related genes and PD-1 resistance-related genes. Monocyte-derived macrophages are a major source of IL-1β production during the immune response to pathogen infection [29, 30]. Previous studies have suggested that IL-1β could promote the invasion and growth of human colon cancer cells [31], and IL-1β polymorphisms are associated with CRC recurrence [32]. IL-1β blockade has been shown to reverse immunosuppression and has been shown to exhibit synergy with PD-1 inhibitors to promote the elimination of several tumor types, including breast [32], pancreatic [18], and renal [33] tumors. Nevertheless, the precise role of IL-1β in CRC immunotherapy has yet to be elucidated. Several studies have suggested that IL-1β can induce the infiltration of MDSCs by producing granulocyte colony stimulating factor, various CXC chemokines, and vascular adhesion molecules [34]. However, the role of IL-1β-mediated MDSC aggregation in CRC immunotherapy remains unclear.

MMP9 is a matrix metalloproteinase that has been identified as a component of the angiogenic switch during carcinogenesis [35, 36]. MMP9 mediates tumor invasion, metastasis, and immune escape through a pro-oncogenic signaling pathway [37–39] and is associated with relapse and poor prognosis in patients with CRC [40]. The combination of MMP9 inhibition with immune checkpoint inhibition enhanced the efficacy of immunotherapy in mouse models of melanoma [41] and breast cancer [42]. MMP9 has also shown promise in the stratification of prognosis and immune checkpoint treatment responsiveness in patients with hepatocellular carcinoma [43]. The present KEGG analysis results for 130 key genes revealed that IL-1β and MMP9 were highly correlated with the MAPK and PI3K-Akt signaling pathways. Recent studies have also indicated that IL-1β upregulates MMP9 expression through different signaling pathways in different diseases [44–46]. The infiltration of MDSCs could further stimulate the secretion of MMP9 [47], and IL-1β-driven accumulation of MDSCs might be one of the main sources of MMP9. However, the function of MMP9 upregulation mediated by IL-1β in CRC immunotherapy needs further study.

In the present study, we described the cellular landscape of immunotherapy-resistant and immunotherapy-sensitive groups at the single-cell level. The degree of aggregation of CD8^+^ T cells and monocytes in the sensitive group was significantly higher than that in the resistant group. IL-1β and MMP9 were identified as the two genes with the highest correlation with anti-PD-1 resistance. Moreover, IL-1β-driven infiltration of MDSCs enhanced anti-PD-1 resistance in MSI-H/dMMR CRC.

## Conclusions

In the present study, the ORR and DCR of MSI-H/dMMR mCRC treated with first-line PD-1 monotherapy were 43.75% (7/16) and 68.75% (11/16), respectively. IL-1β and CD8^+^ T cells were found to have the highest correlation with anti-PD-1 resistance among genes and cell types, respectively. Mouse experiments demonstrated that IL-1β-driven MDSC infiltration suppressed the accumulation of CD8^+^ T cells, which enhanced the anti-PD-1 resistance of MSI-H/dMMR CRC. Together, these findings suggest that IL-1β antagonists may prove promising as new drugs to reverse resistance to PD-1 inhibitors.

## Supplementary Information

**Additional file 1:** **Table S1.** Single cell sequencing data of clinical samples from 6 patients with MSI-H/dMMR.

**Additional file 2:** **Table S2.** Markers used in cell subtype analysis pipeline.

**Additional file 3: Table S3.** Number of cells of 9 cell subtypes in each sample.

**Additional file 4:** **Figure S1.** GO and KEGG analysis of marker genes.

**Additional file 5:** **Table S4.** 1623 differentially expressed genes in 9 cell subsets.

**Additional file 6:** **Table S5.** 1454 pseudotime-related genes were identified from 1623 feature genes.

**Additional file 7:** **Figure S2.** KEGG and GO analysis of pseudotime-related genes.

**Additional file 8:** **Table S6.** 155 DEGs in gene expression of each cell subtype between sensitive group and resistant group.

**Additional file 9:** **Figure S3.** GO and KEGG analysis of PD-1 resistance-related DEGs.

**Additional file 10:** **Table S7.** 130 common genes among 454 pseudo-time related genes and 155 (de- duplication) PD-1 resistance related genes.

**Additional file 11:** **Table S8.** VarElect analyse of 130 common genes.

**Additional file 12:** **Figure S4.** VarElect analysis results of 130 common genes in 454 pseudotime-related genes and 155 (de-duplication) PD-1 resistance related genes DEGs. **Figure S5.** (A) The cytokine‒cytokine receptor interaction and (B) the MAPK and PI3K-Akt signaling pathway maps of IL-1β and MMP9. **Figure S6.** Construction of stable CT26 cell line overexpressing IL-1β. (A) Colorectal cancer cell line CT26 used in this experiment. (B) Plasmid map of IL-1β overexpression vector. (C) Screening positive colonies by ampicillin after plasmid transformation. (D) Gel map of plasmid electrophoresis. (E) Western blotting was applied to detect the expression of IL-1β in stably transformed cell lines. (F) qPCR was used to detect the expression of IL-1β in stably transformed cell lines.

**Additional file 13.** Images of the original blots.

## Data Availability

The scRNA-seq datasets generated and analysed during the current study are available in the NCBI repository [https://www.ncbi.nlm.nih.gov/sra/PRJNA932556].

## Abbreviations

BP: Biological process
CC: Cell composition
CR: Complete response
CRC: Colorectal cancer
DCR: Disease control rate
DCs: Dendritic cells
DEGs: Differentially expressed genes
dMMR: Deficient mismatch repair
IHC: Immunohistochemistry
IF: Immunofluorescence
MDSCs: Myeloid-derived suppressor cells
mCRC: Metastatic colorectal cancer
M-MDSCs: Monocytic myeloid-derived suppressor cells
MSI-H: Microsatellite instability-high
MSS: Microsatellite stable
ORR: Objective response rate
PD: Progressive disease
PD-1: Programmed cell death protein-1
PMN-MDSCs: Polymorphonuclear myeloid-derived suppressor cells
PR: Partial response
PPI: Perform protein–protein interaction
MF: Molecular function
scRNA-seq: Single-cell RNA sequencing
SD: Stable disease
STRING: Search Tool for the Retrieval of Interacting Genes
TME: Tumor microenvironment
t-SNE: T-distributed stochastic neighbor embedding
