# Supplementary material for: Single-cell sequencing reveals the immune microenvironment landscape related to anti-PD-1 resistance in metastatic colorectal cancer with high microsatellite instability
Source: BMC Med. 2023 Apr 27;21:161. doi: 10.1186/s12916-023-02866-y (PMC10142806; doi:10.1186/s12916-023-02866-y)
Supplement: Supplementary file 4 — Additional file 4: Figure S1. GO and KEGG analysis of marker genes. [file 12916_2023_2866_MOESM4_ESM.pptx]

## Slide 1
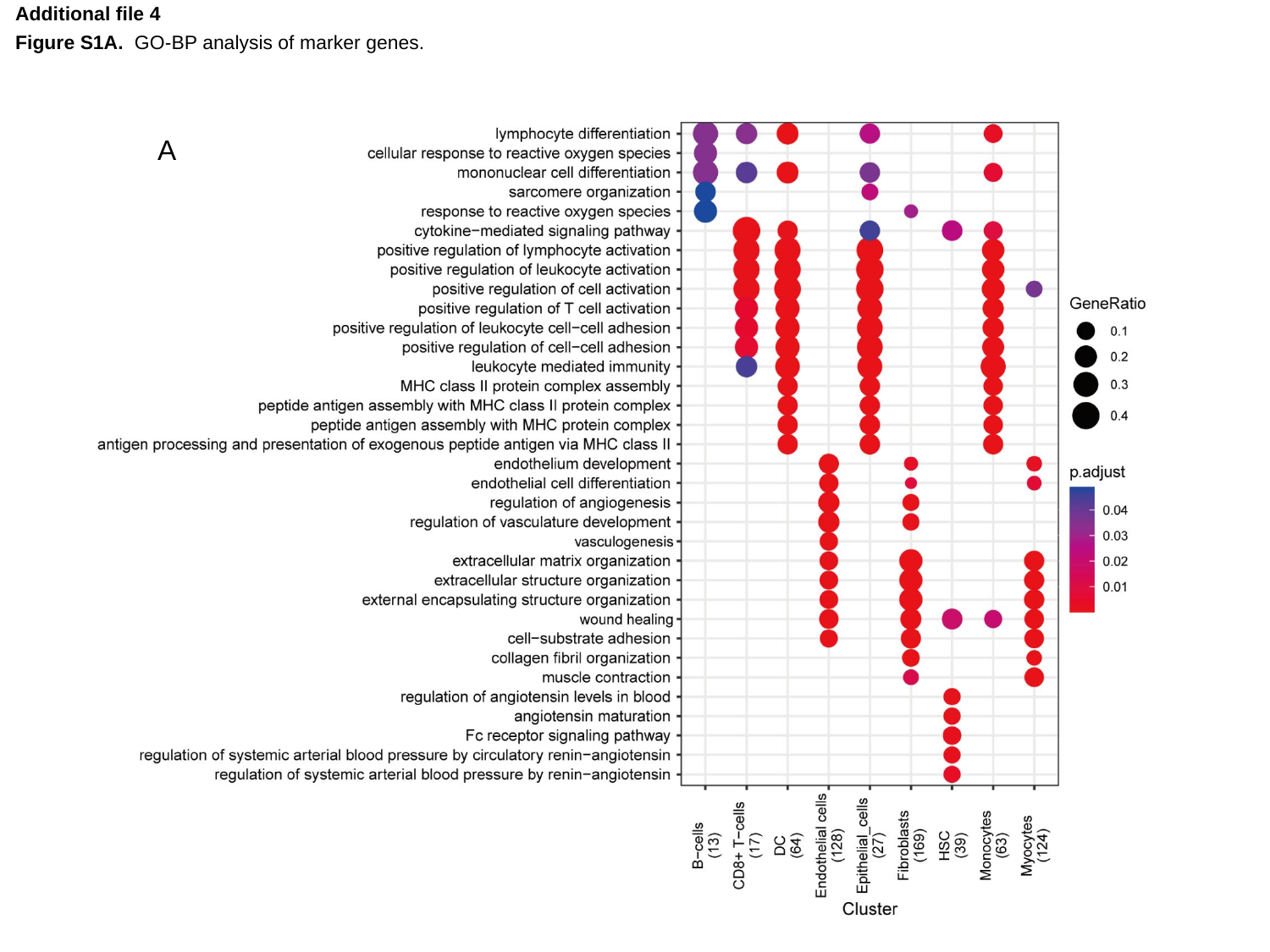

Additional file 4
Figure S1A. GO-BP analysis of marker genes.
A

## Slide 2
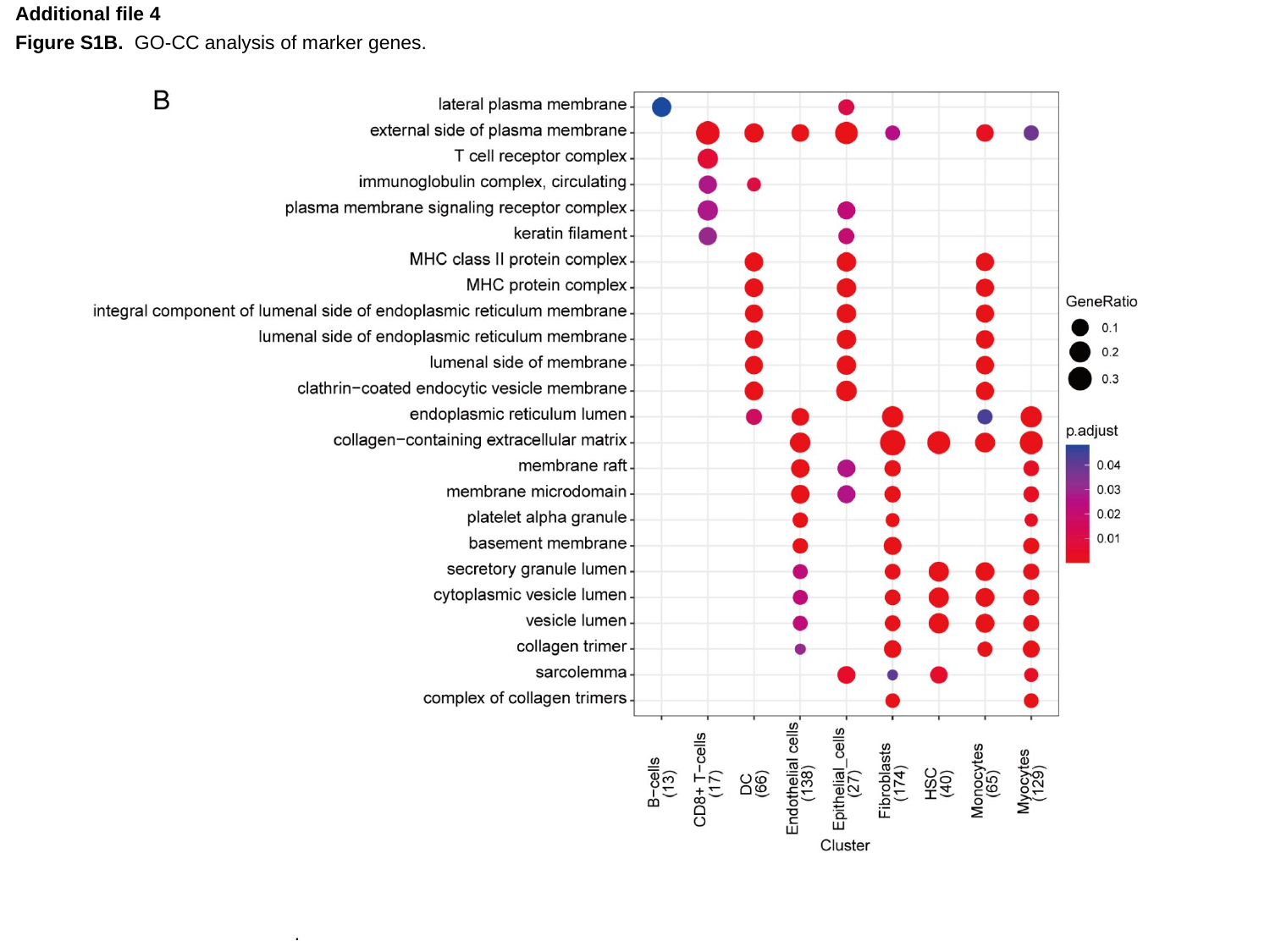

Additional file 4
Figure S1B. GO-CC analysis of marker genes.
.

## Slide 3
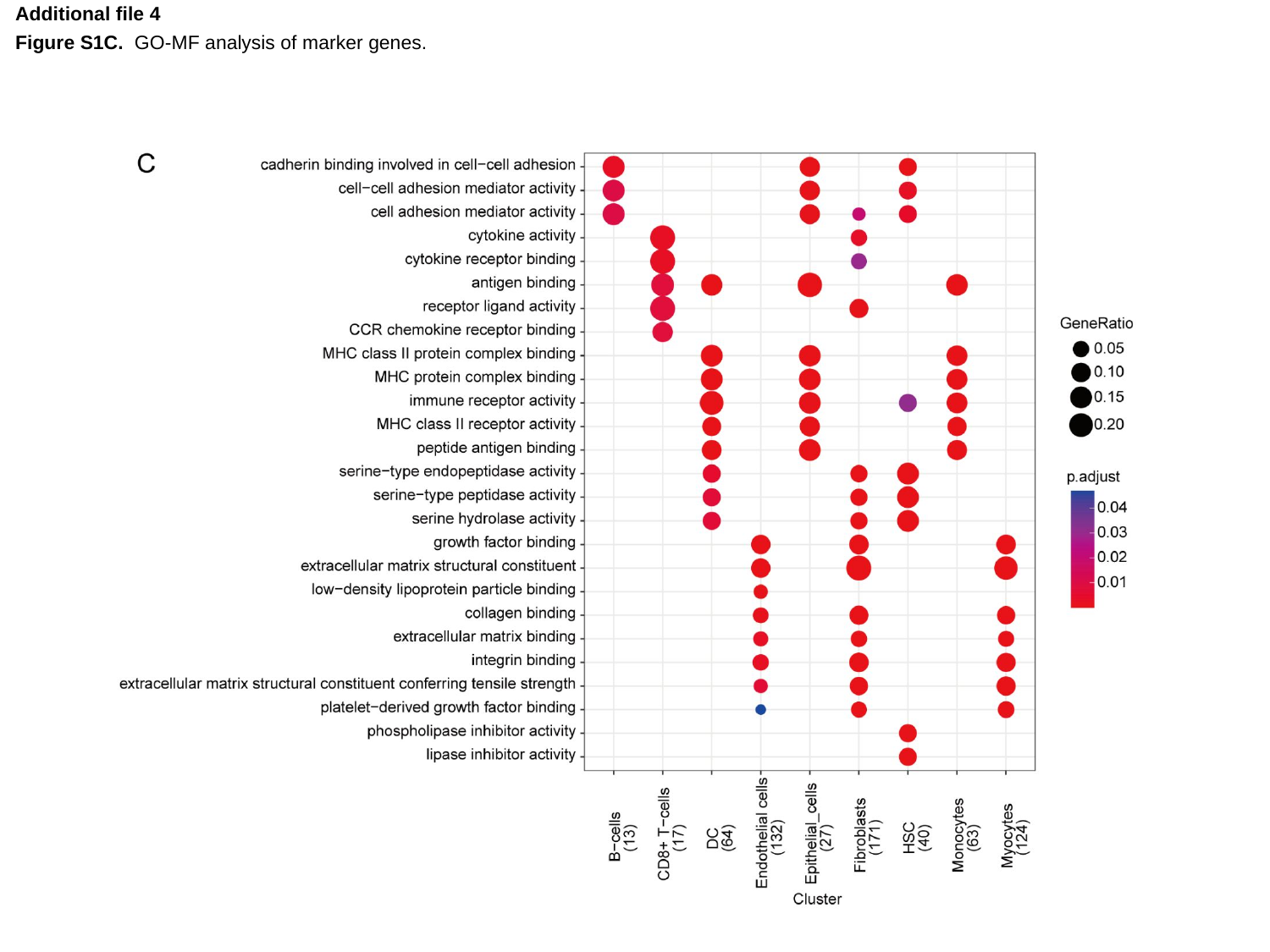

Additional file 4
Figure S1C. GO-MF analysis of marker genes.

## Slide 4
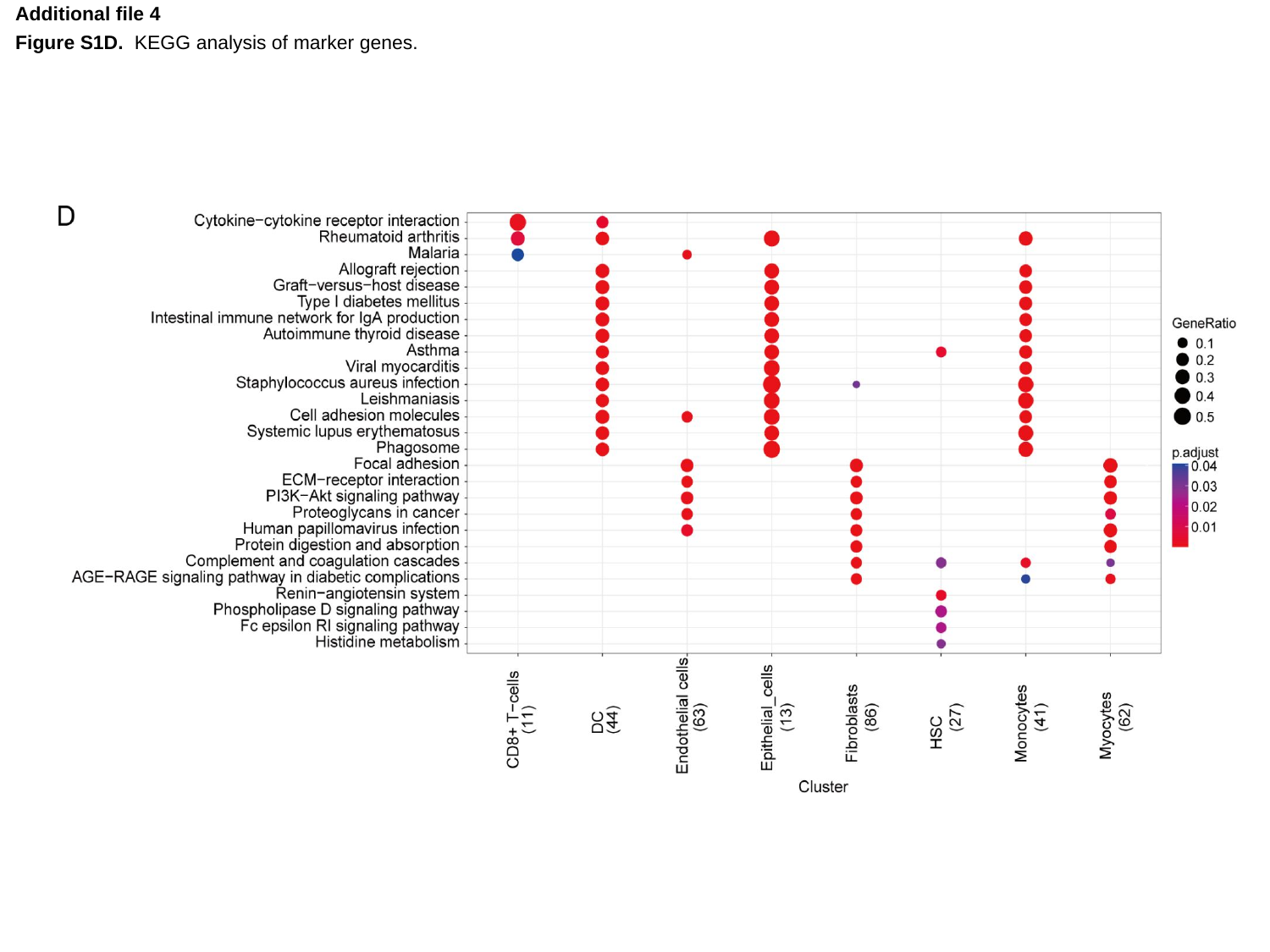

Additional file 4
Figure S1D. KEGG analysis of marker genes.
