# Supplementary material for: Single-cell sequencing reveals the immune microenvironment landscape related to anti-PD-1 resistance in metastatic colorectal cancer with high microsatellite instability
Source: BMC Med. 2023 Apr 27;21:161. doi: 10.1186/s12916-023-02866-y (PMC10142806; doi:10.1186/s12916-023-02866-y)
Supplement: Supplementary file 7 — Additional file 7: Figure S2. KEGG and GO analysis of pseudotime-related genes. [file 12916_2023_2866_MOESM7_ESM.pptx]

## Slide 1
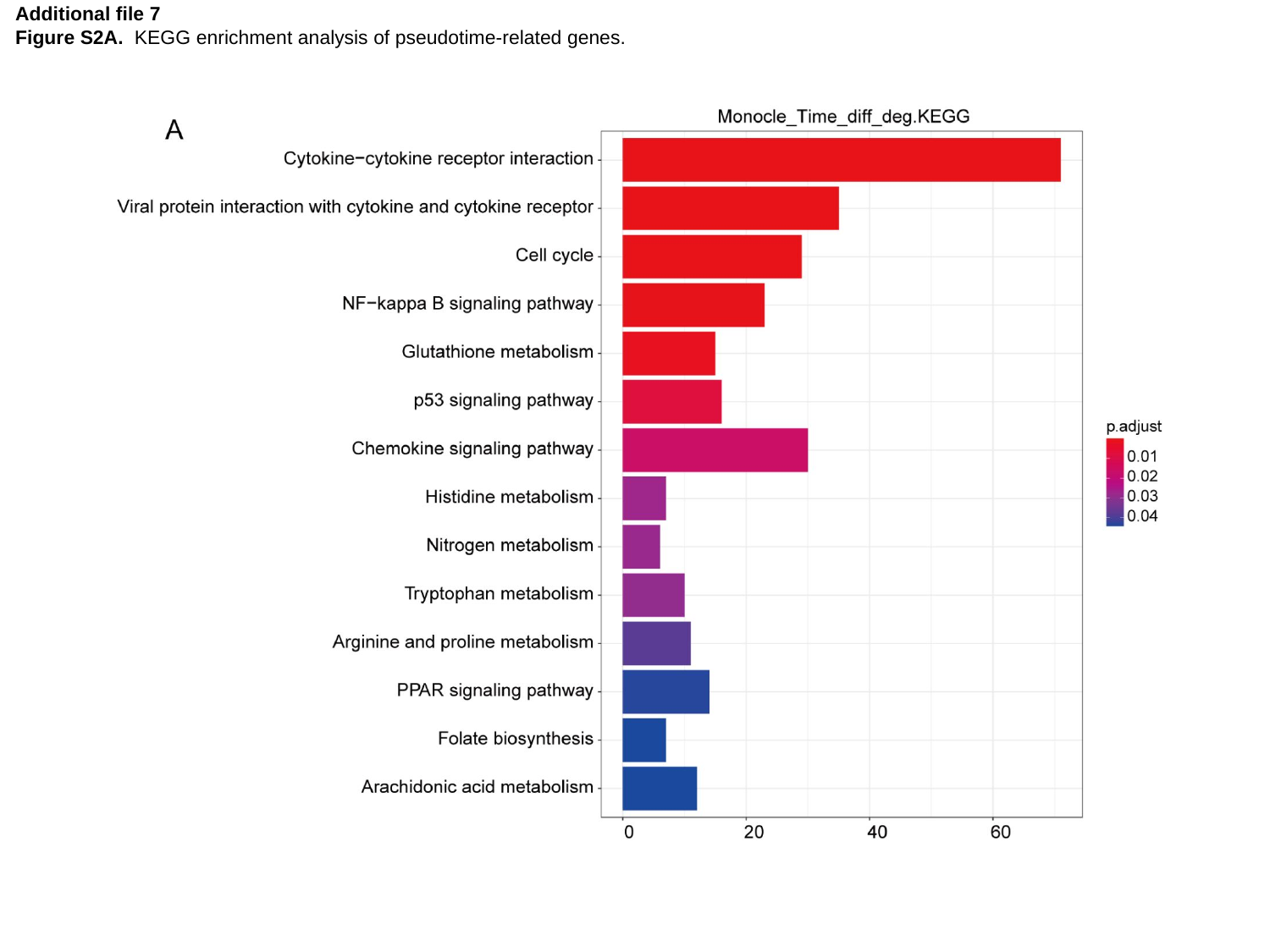

Additional file 7
Figure S2A. KEGG enrichment analysis of pseudotime-related genes.

## Slide 2
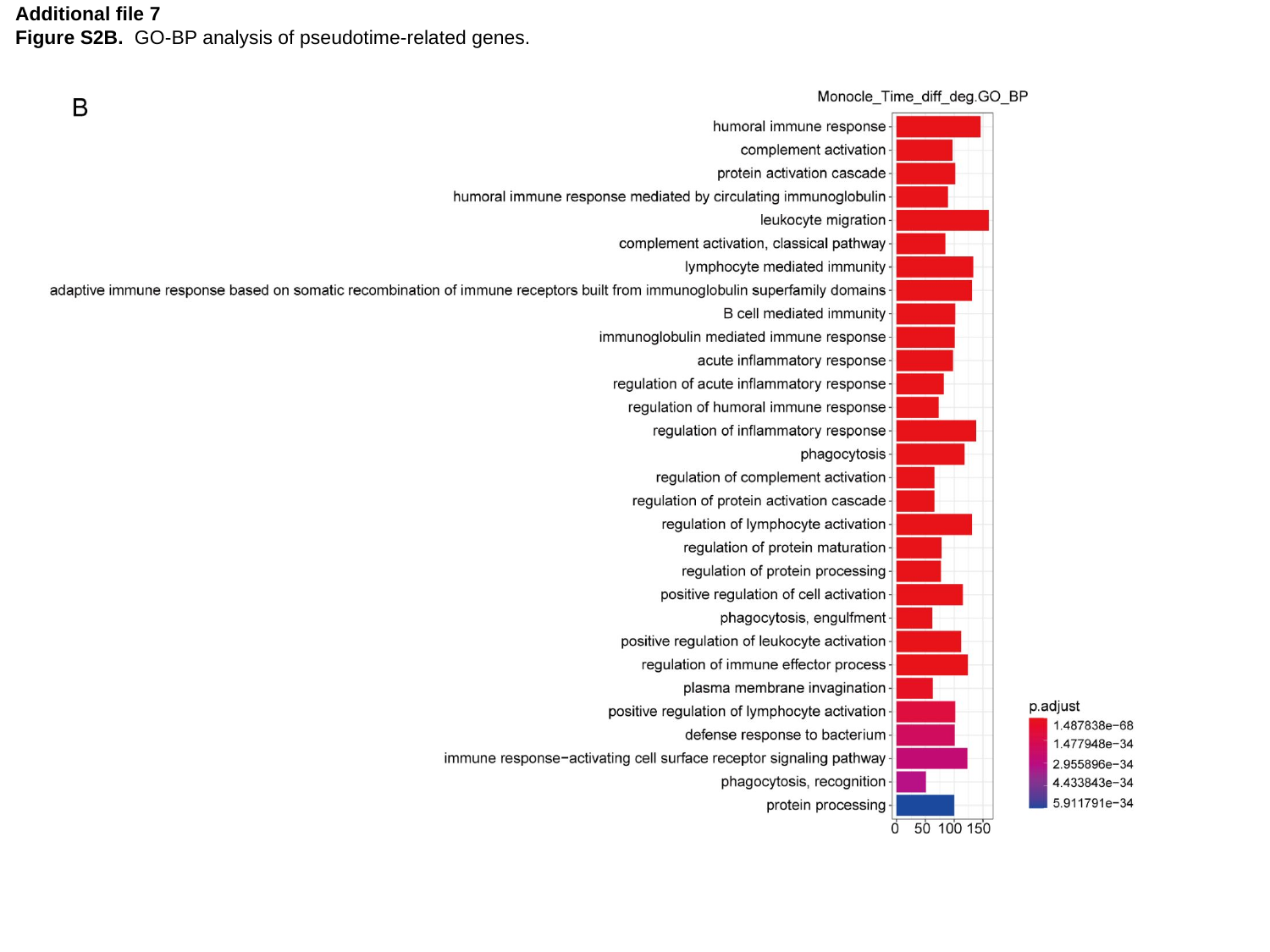

Additional file 7
Figure S2B. GO-BP analysis of pseudotime-related genes.

## Slide 3
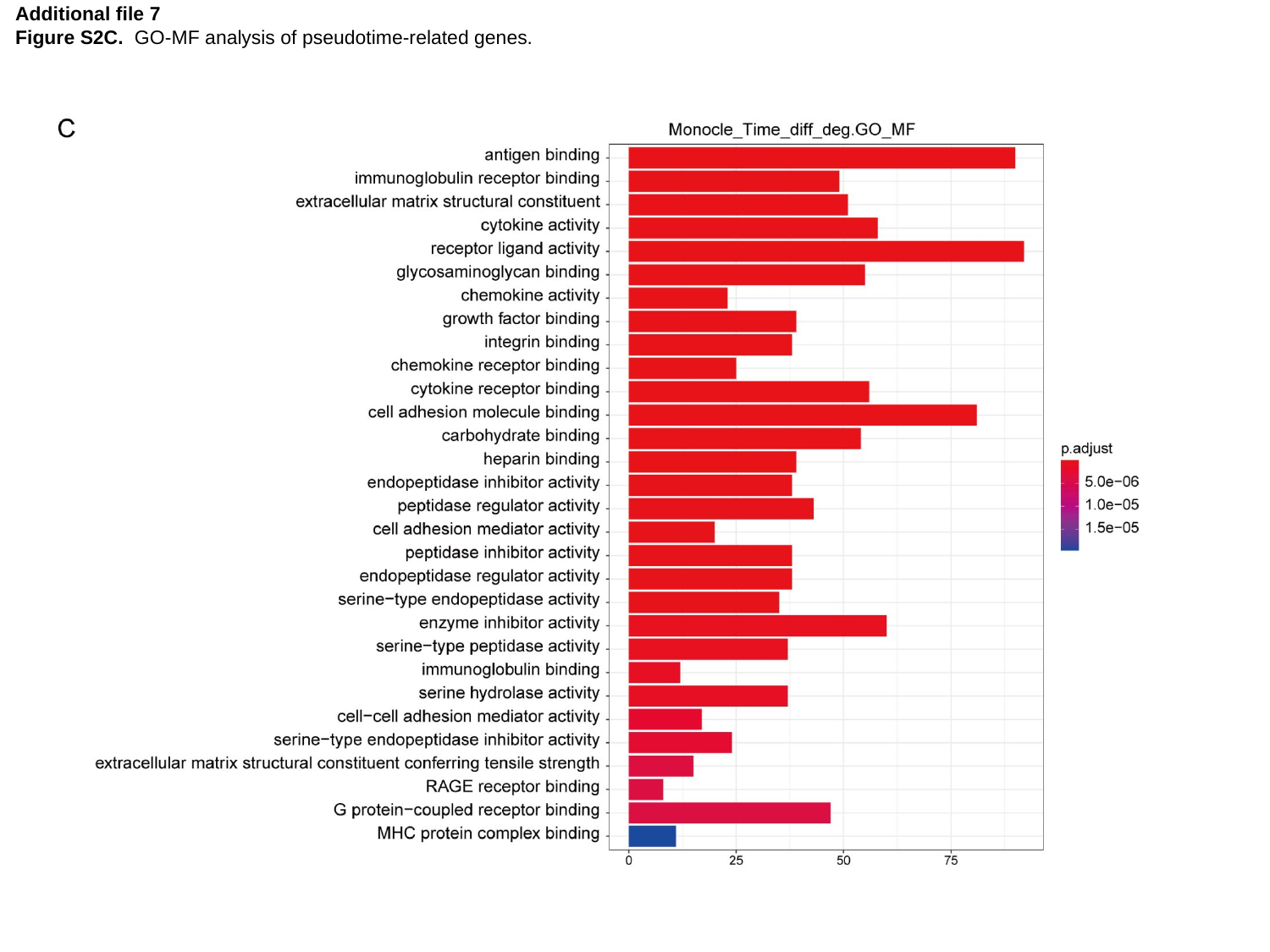

Additional file 7
Figure S2C. GO-MF analysis of pseudotime-related genes.

## Slide 4
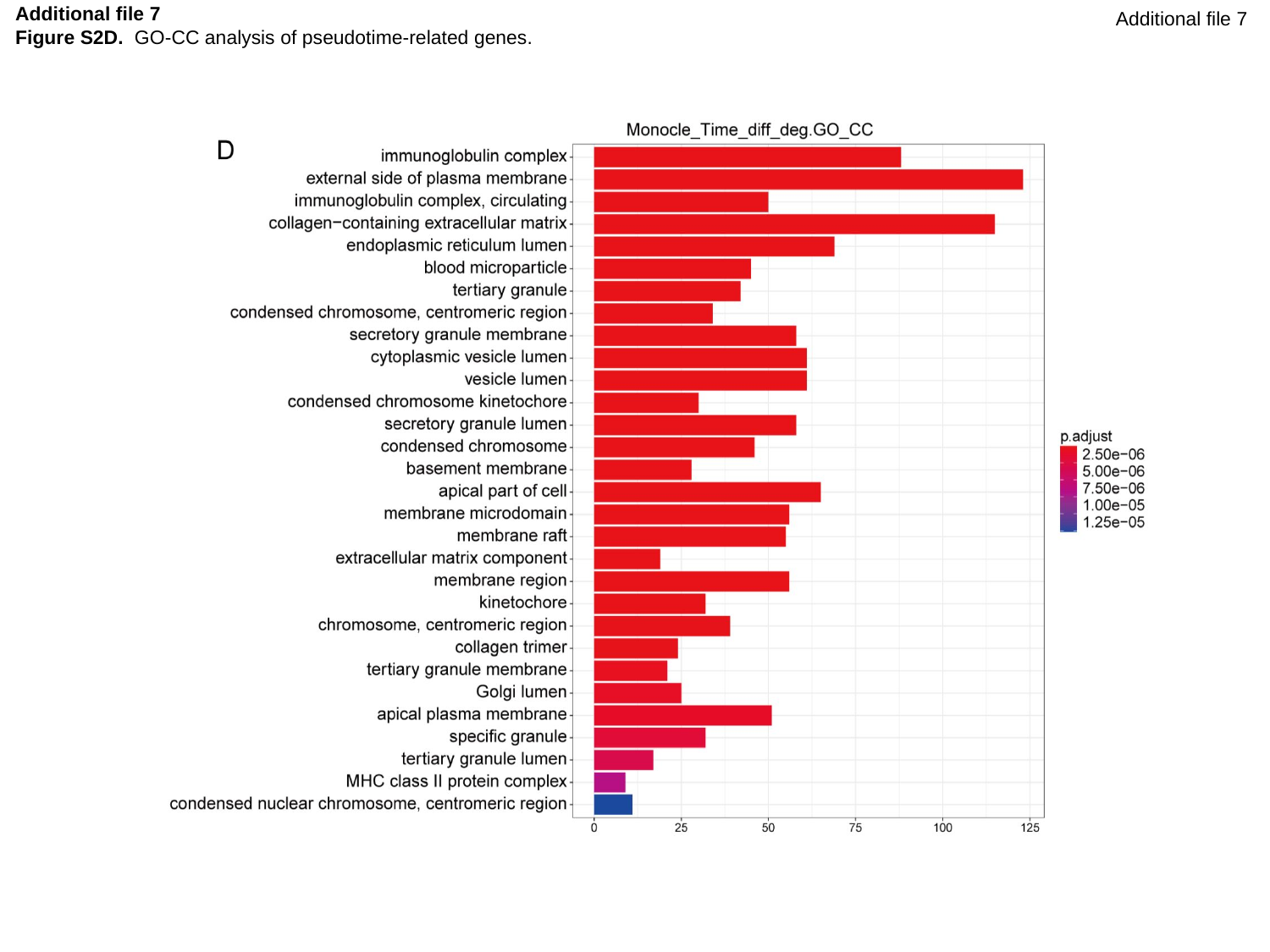

Additional file 7
Additional file 7
Figure S2D. GO-CC analysis of pseudotime-related genes.
