# Supplementary material for: Single-cell sequencing reveals the immune microenvironment landscape related to anti-PD-1 resistance in metastatic colorectal cancer with high microsatellite instability
Source: BMC Med. 2023 Apr 27;21:161. doi: 10.1186/s12916-023-02866-y (PMC10142806; doi:10.1186/s12916-023-02866-y)
Supplement: Supplementary file 9 — Additional file 9: Figure S3. GO and KEGG analysis of PD-1 resistance-related DEGs. [file 12916_2023_2866_MOESM9_ESM.pptx]

## Slide 1
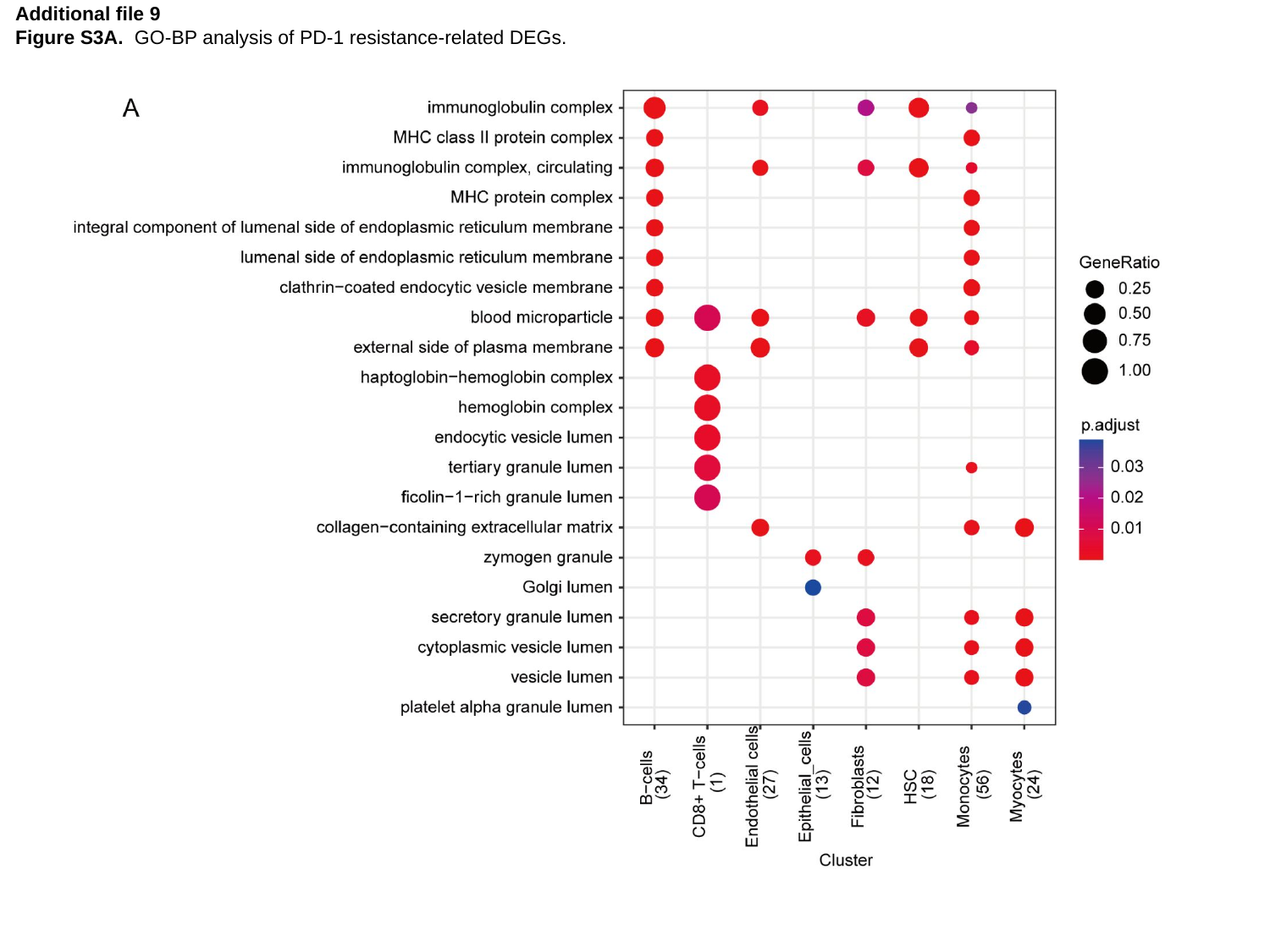

Additional file 9
Figure S3A. GO-BP analysis of PD-1 resistance-related DEGs.

## Slide 2
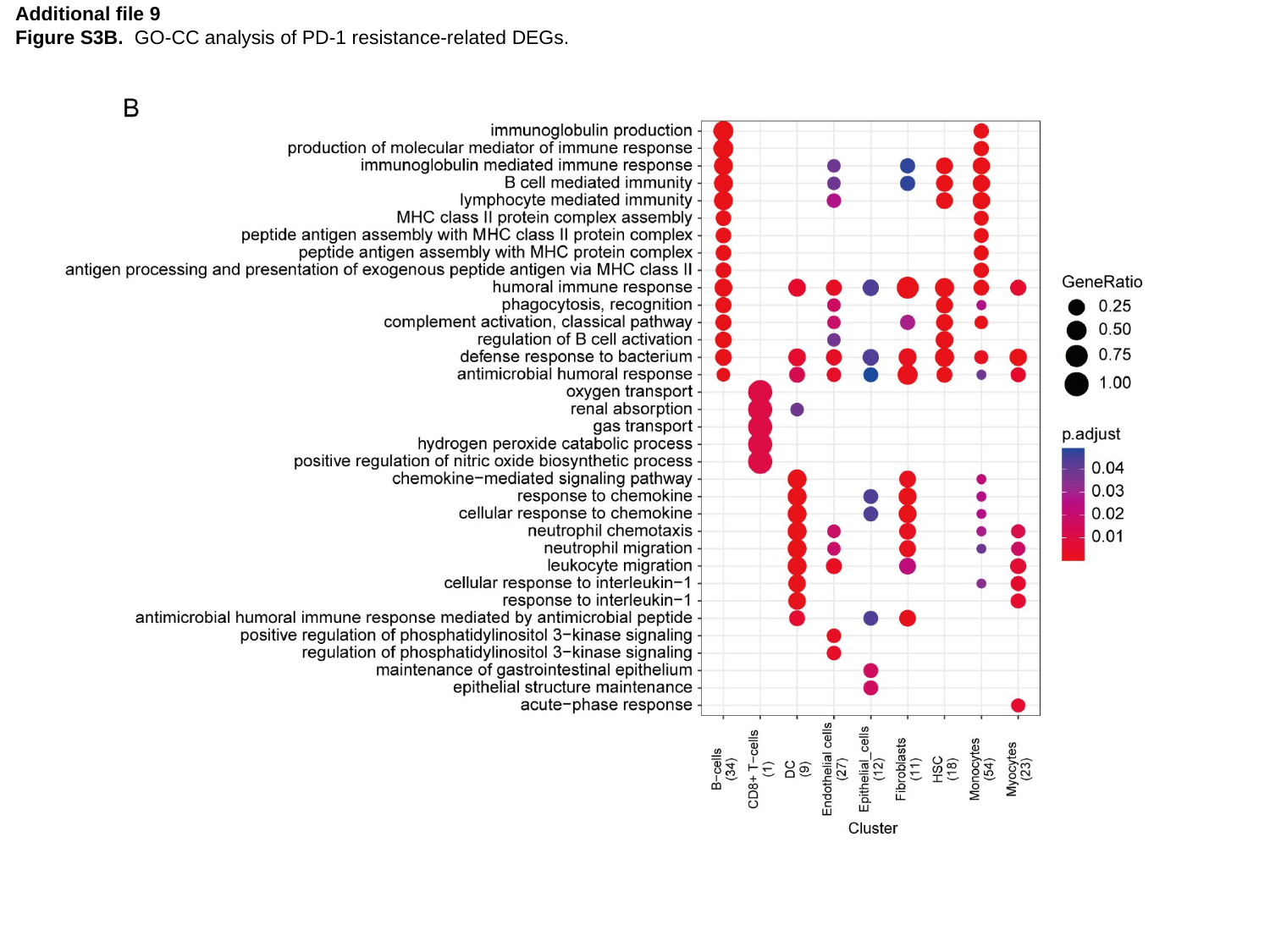

Additional file 9
Figure S3B. GO-CC analysis of PD-1 resistance-related DEGs.

## Slide 3
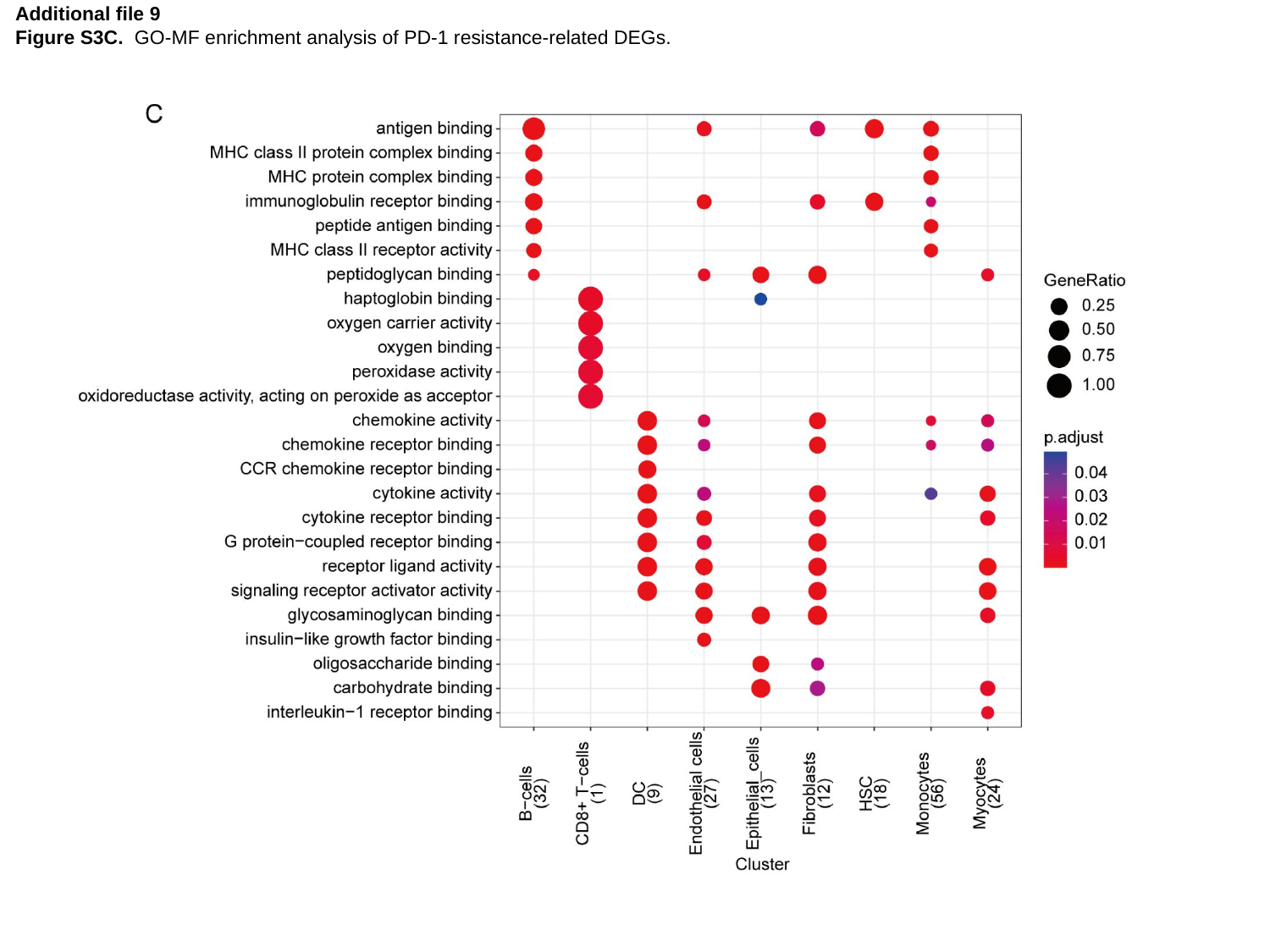

Additional file 9
Figure S3C. GO-MF enrichment analysis of PD-1 resistance-related DEGs.

## Slide 4
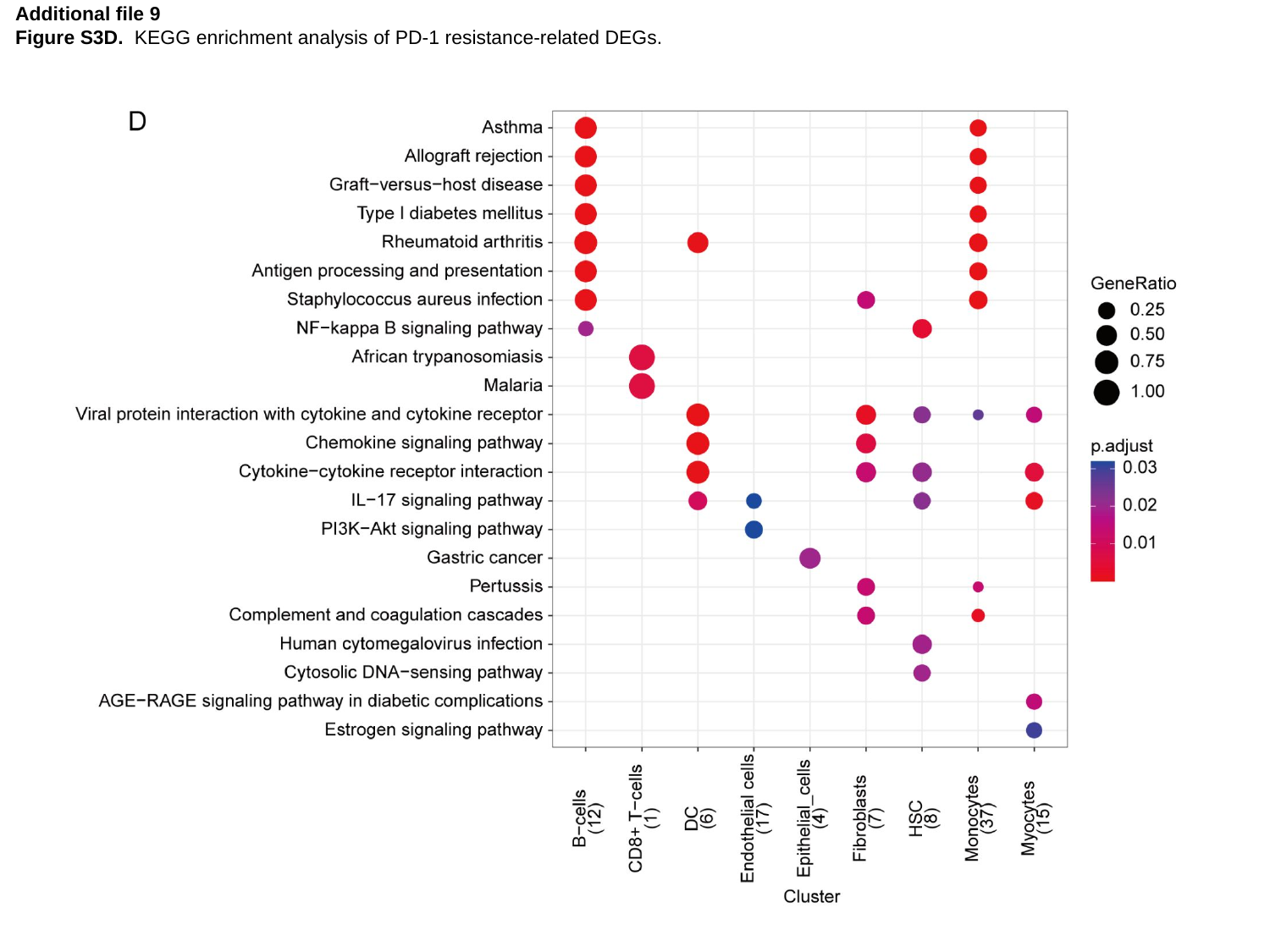

Additional file 9
Figure S3D. KEGG enrichment analysis of PD-1 resistance-related DEGs.
