# Supplementary material for: Single-cell sequencing reveals the immune microenvironment landscape related to anti-PD-1 resistance in metastatic colorectal cancer with high microsatellite instability
Source: BMC Med. 2023 Apr 27;21:161. doi: 10.1186/s12916-023-02866-y (PMC10142806; doi:10.1186/s12916-023-02866-y)

Additional file 12

Figure S4. VarElect analysis results of 130 common genes in 454 pseudotime-related genes and 155 (de-duplication) PD-1 resistance related genes DEGs.

#	Symbol	Description	Type	Matched Phenotypes	Matched Phenotypes Count	Score	-Log10(p)	Average Disease Causing Likelihood
1	IL1B	Interleukin 1 Beta	Protein	"colorectal cancer"; caner	2	17.72	2.36	81.8
2	MMP9	Matrix Metallopeptidase 9	Protein	"colorectal cancer"; caner	2	13.45	2.28	20.3
3	CEACAM5	CEA Cell Adhesion Molecule 5	Protein	"colorectal cancer"; caner	2	12.25	2.25	21.5
4	PTGS2	Prostaglandin-Endoperoxide Synthase 2	Protein	"colorectal cancer"	1	11.32	2.33	66.5
5	IL1RN	Interleukin 1 Receptor Antagonist	Protein	"colorectal cancer"; caner	2	10.84	2.16	79.1
6	MUC2	Mucin 2, Oligomeric Mucus/Gel-Forming	Protein	"colorectal cancer"; caner	2	10.52	2.14	ND
7	SPP1	Secreted Phosphoprotein 1	Protein	"colorectal cancer"; caner	2	9.43	2.01	24.4
8	IGFBP3	Insulin Like Growth Factor Binding Protein 3	Protein	"colorectal cancer"	1	9.23	2.27	30.3
9	KRT19	Keratin 19	Protein	"colorectal cancer"; caner	2	8.13	1.77	48.7
10	REG4	Regenerating Family Member 4	Protein	"colorectal cancer"; caner	2	7.53	1.63	53.7
11	STMN1	Stathmin 1	Protein	"colorectal cancer"; caner	2	7.36	1.58	60.0
12	FN1	Fibronectin 1	Protein	"colorectal cancer"	1	6.09	1.88	56.9
13	MMP12	Matrix Metallopeptidase 12	Protein	"colorectal cancer"	1	5.7	1.76	ND
14	EREG	Epiregulin	Protein	"colorectal cancer"	1	5.69	1.76	67.0
15	ENG	Endoglin	Protein	"colorectal cancer"	1	5.5	1.69	58.1
16	LGALS3	Galectin 3	Protein	"colorectal cancer"	1	5.48	1.69	22.7
17	F3	Coagulation Factor III, Tissue Factor	Protein	"colorectal cancer"	1	5.24	1.59	45.4
18	CXCL1	C-X-C Motif Chemokine Ligand 1	Protein	"colorectal cancer"	1	5.21	1.58	60.0
19	S100A9	S100 Calcium Binding Protein A9	Protein	"colorectal cancer"	1	5.15	1.56	67.3
20	CCL20	C-C Motif Chemokine Ligand 20	Protein	"colorectal cancer"	1	5.12	1.55	64.2

Additional file 12

Figure S5. The cytokine–cytokine receptor interaction and the MAPK and PI3K-Akt signaling pathway maps of IL-1β and MMP9.

A

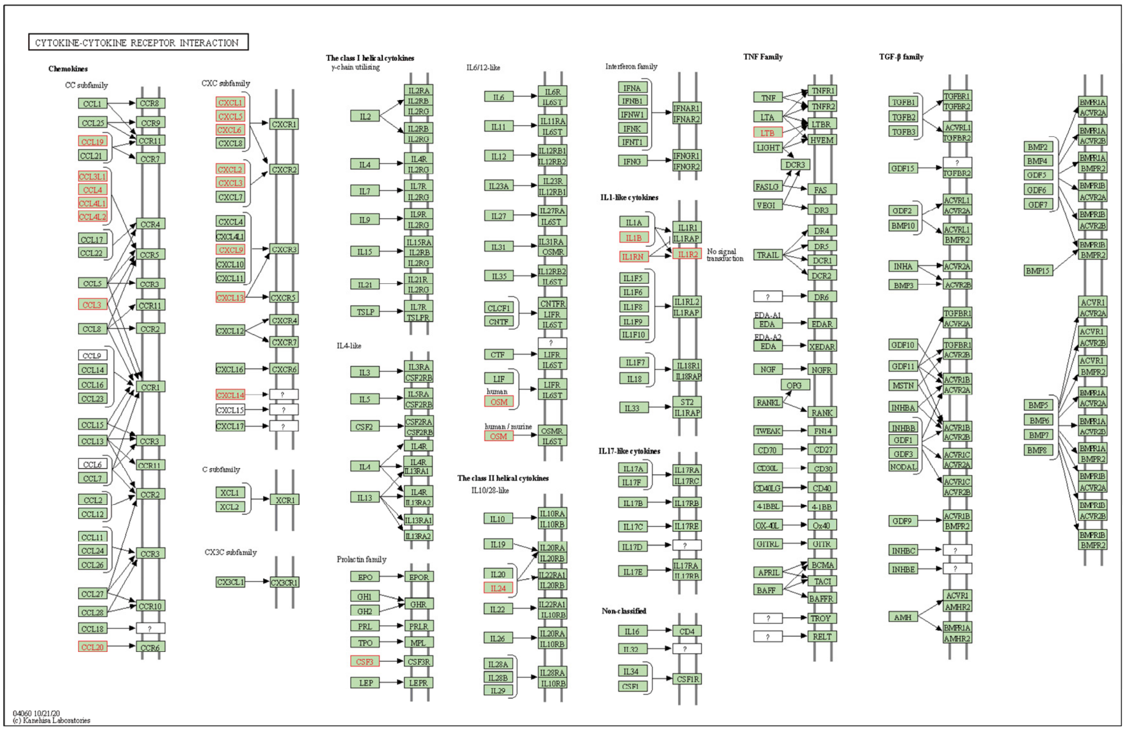

B

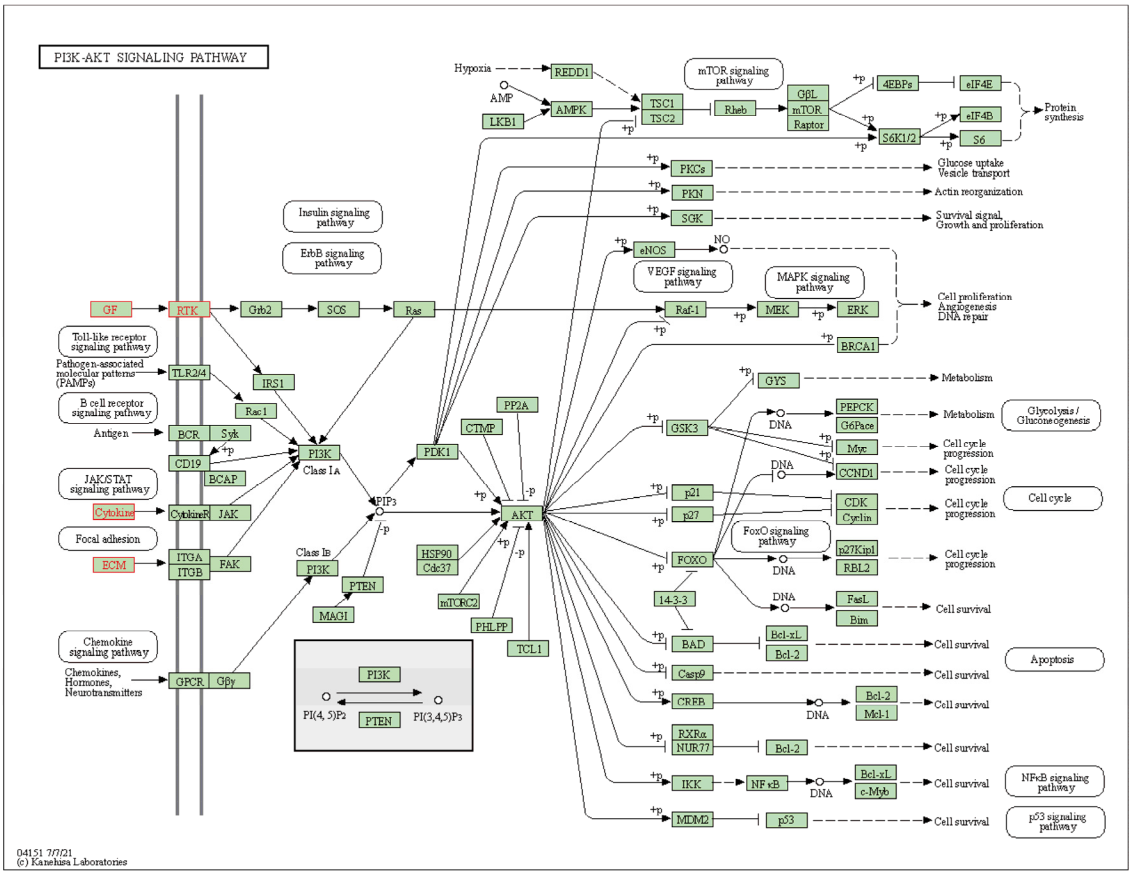

Additional file 12

Figure S6. Construction of stable CT26 cell line overexpressing IL-1β.

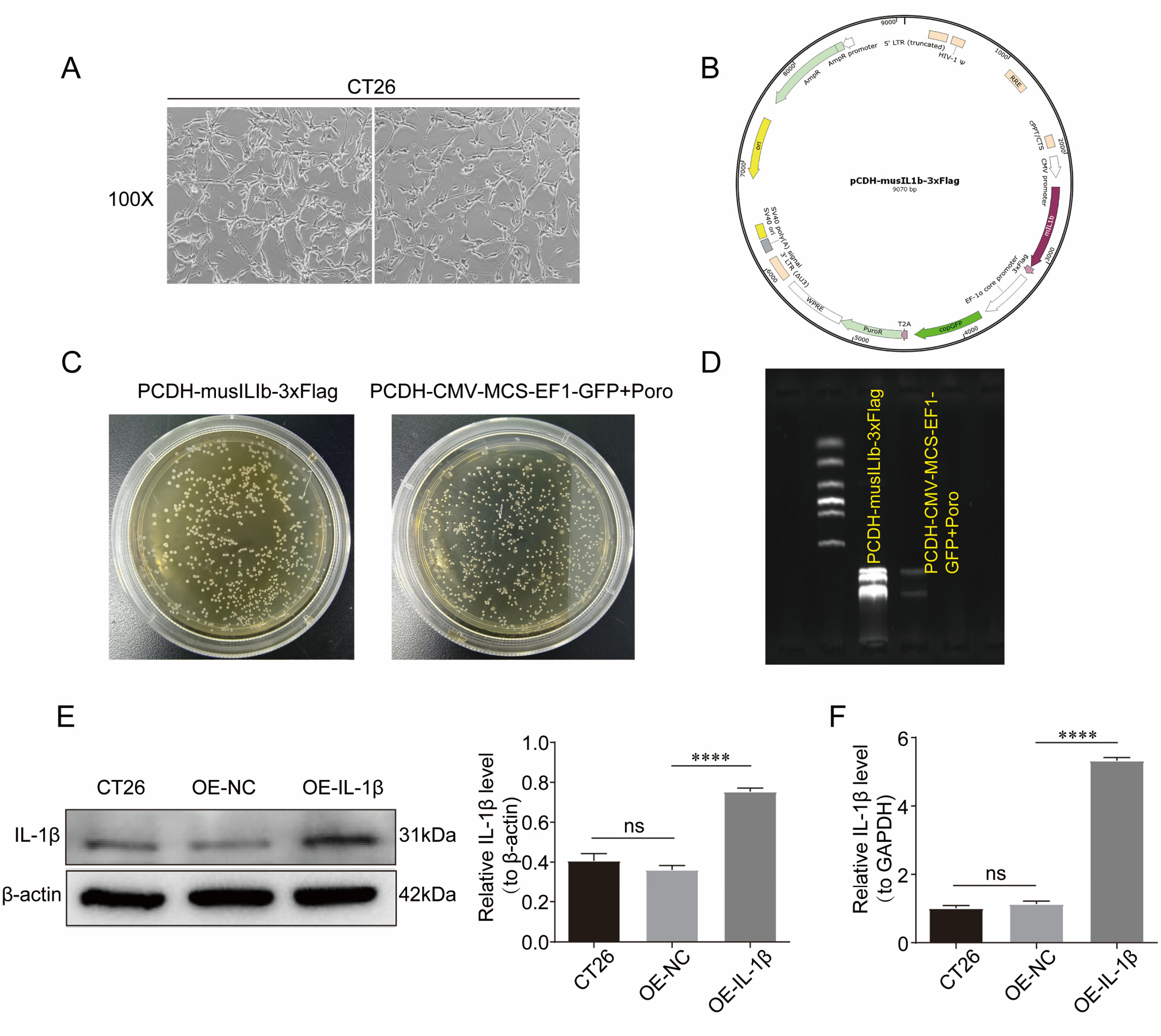

Supplement: Supplementary file 12 — Additional file 12: Figure S4. VarElect analysis results of 130 common genes in 454 pseudotime-related genes and 155 (de-duplication) PD-1 resistance related genes DEGs. Figure S5. (A) The cytokine‒cytokine receptor interaction and (B) the MAPK and PI3K-Akt signaling pathway maps of IL-1β and MMP9. Figure S6. Construction of stable CT26 cell line overexpressing IL-1β. (A) Colorectal cancer cell line CT26 used in this experiment. (B) Plasmid map of IL-1β overexpression vector. (C) Screening positive colonies by ampicillin after plasmid transformation. (D) Gel map of plasmid electrophoresis. (E) Western blotting was applied to detect the expression of IL-1β in stably transformed cell lines. (F) qPCR was used to detect the expression of IL-1β in stably transformed cell lines. [file 12916_2023_2866_MOESM12_ESM.pdf]
