# Supplementary material for: Single-cell sequencing reveals the immune microenvironment landscape related to anti-PD-1 resistance in metastatic colorectal cancer with high microsatellite instability
Source: BMC Med. 2023 Apr 27;21:161. doi: 10.1186/s12916-023-02866-y (PMC10142806; doi:10.1186/s12916-023-02866-y)
Supplement: Supplementary file 13 — Additional file 13. Images of the original blots. [file 12916_2023_2866_MOESM13_ESM.pptx]

## Slide 1
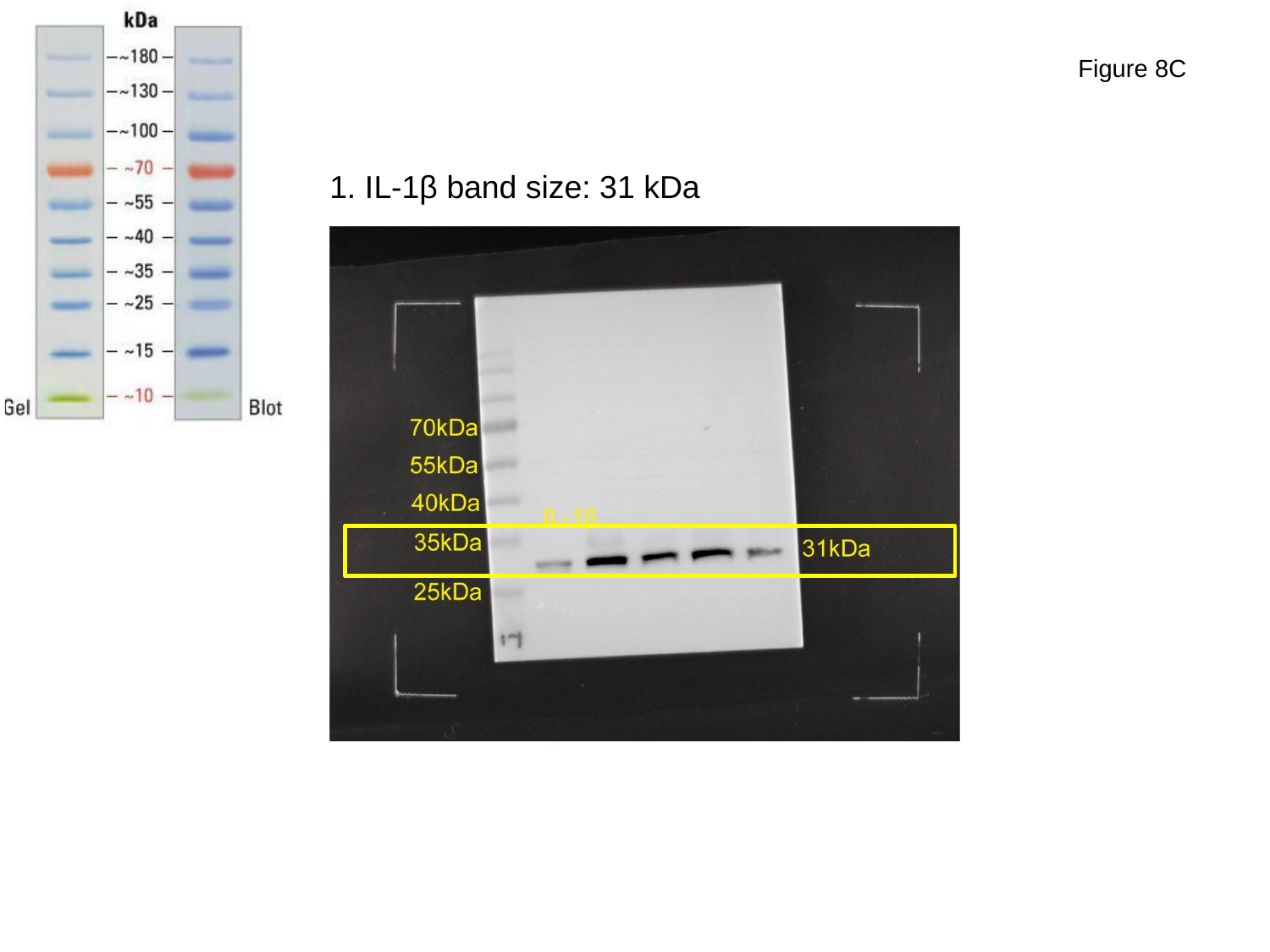

Figure 8C
1. IL-1β band size: 31 kDa

## Slide 2
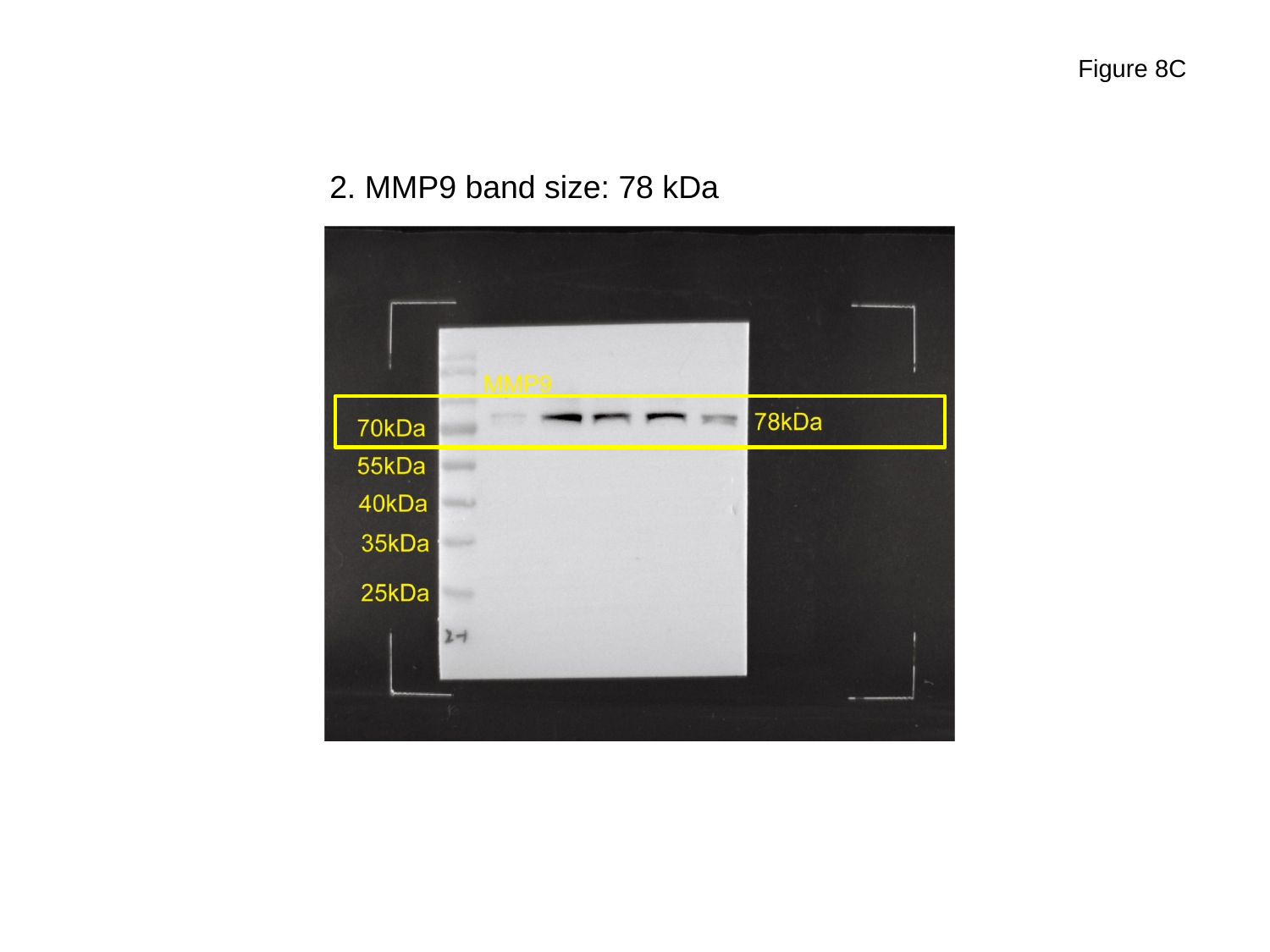

Figure 8C
2. MMP9 band size: 78 kDa

## Slide 3
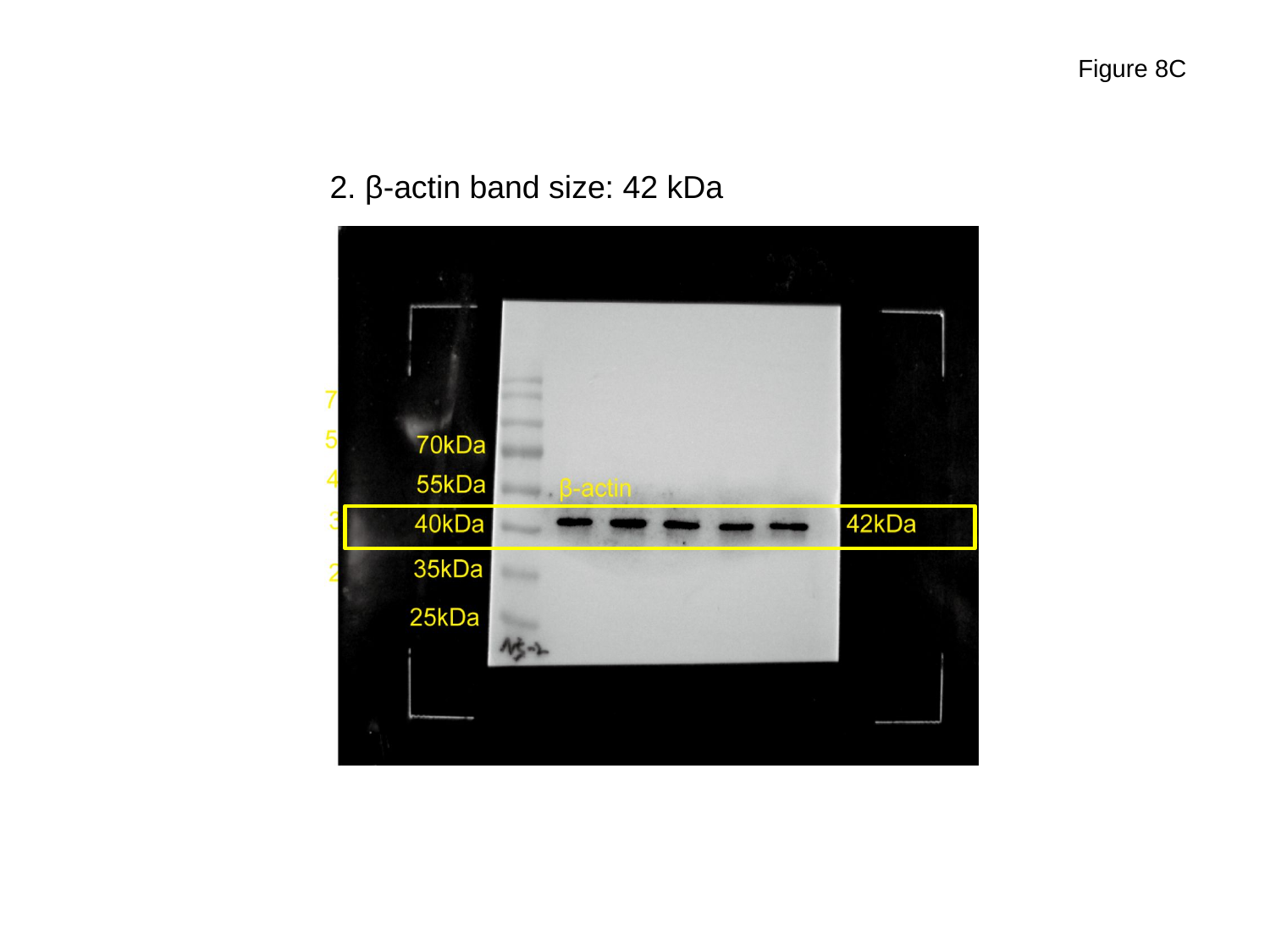

Figure 8C
2. β-actin band size: 42 kDa

## Slide 4
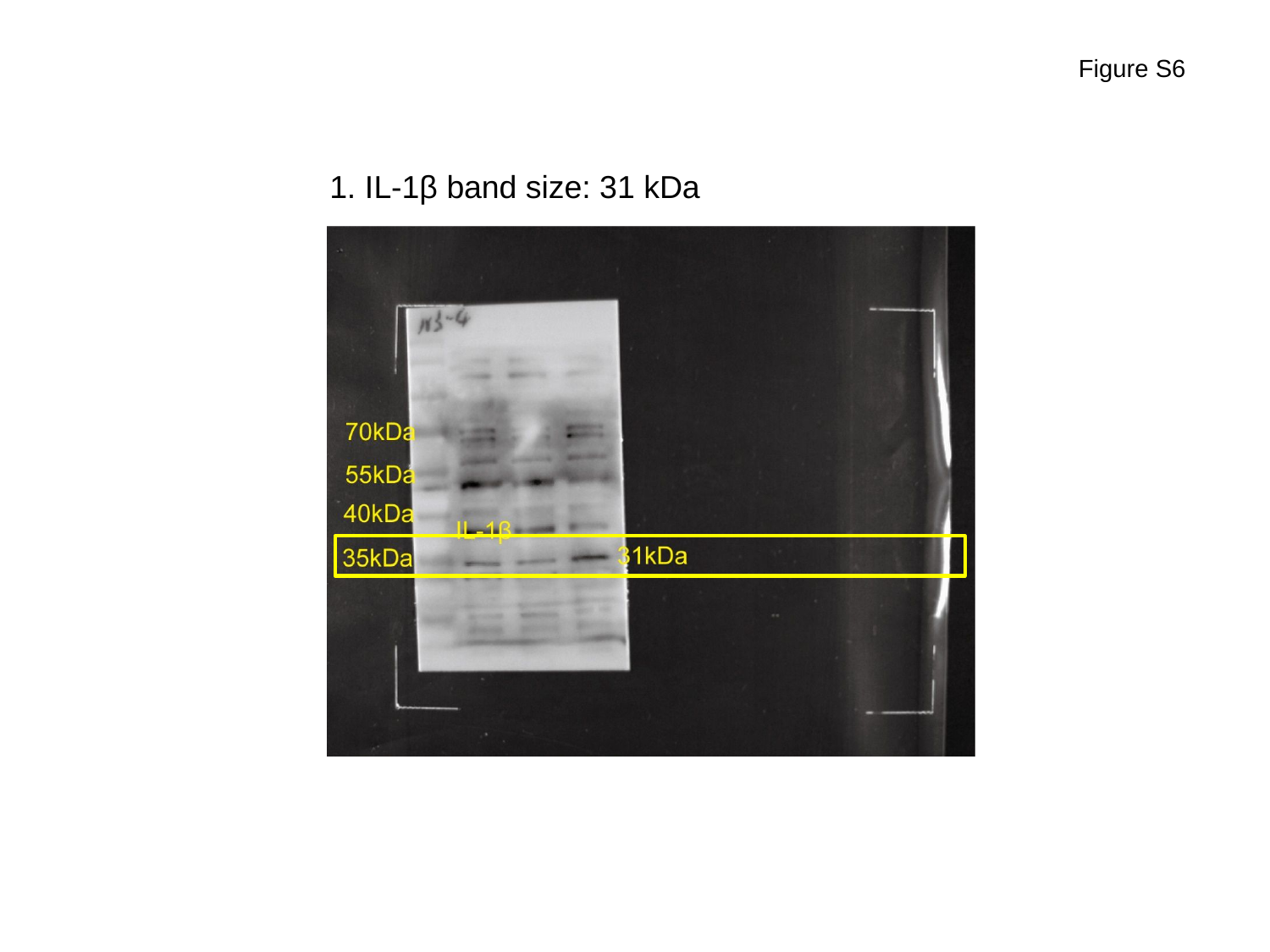

Figure S6
1. IL-1β band size: 31 kDa

## Slide 5
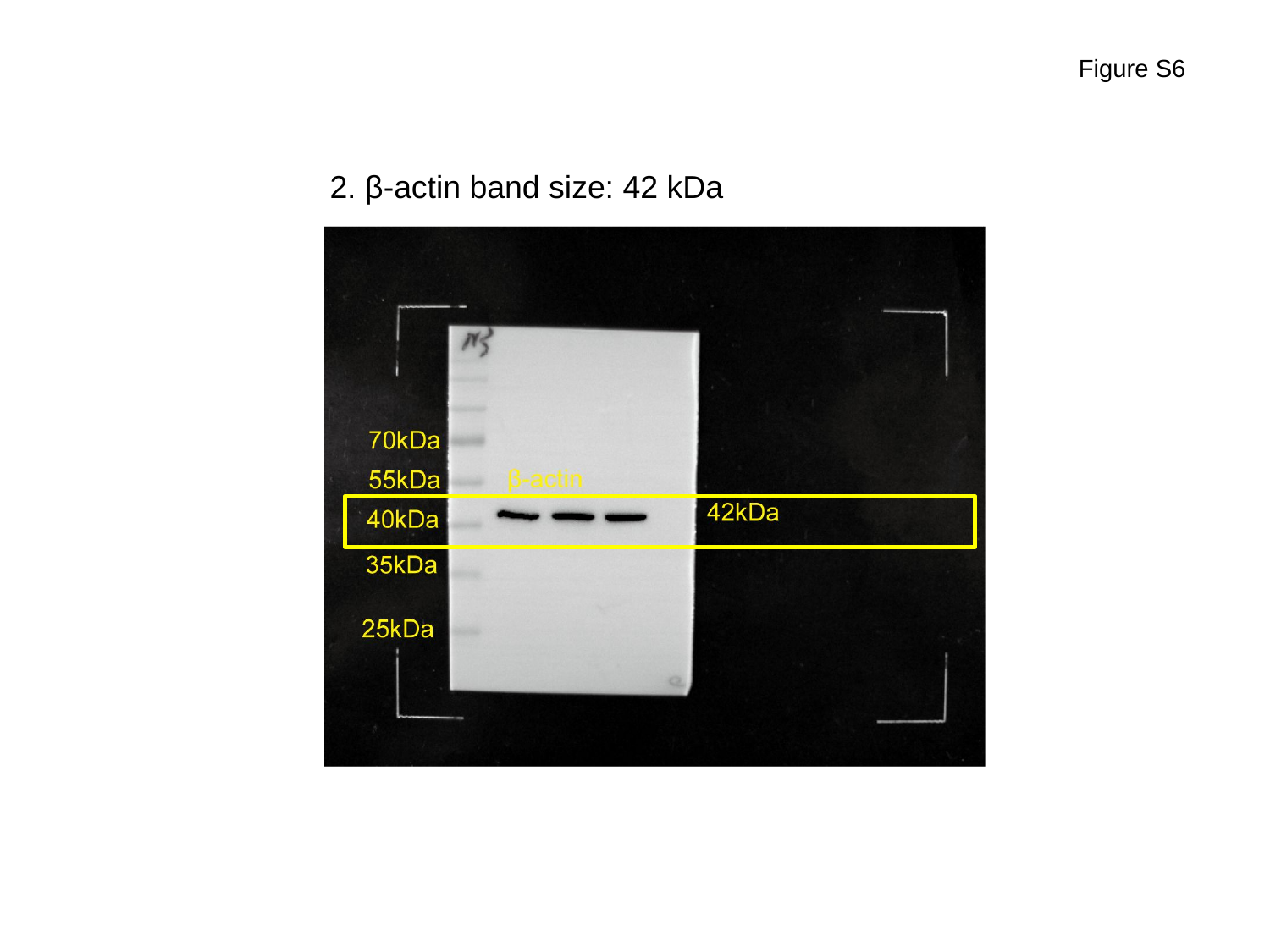

Figure S6
2. β-actin band size: 42 kDa
